# Efficacy and safety of Songjiao Dihuang Tang decoction for the dynamic/adaptive treatment of immune checkpoint inhibitor-associated myocarditis: study protocol and statistical analysis plan for a stop&go, multicentre, randomized, parallel-controlled, double-blind, superiority clinical trial

**DOI:** 10.3389/fphar.2026.1797368

**Published:** 2026-05-12

**Authors:** Jingyi Long, Jiabin Zheng, Zihan Jin, Jie Zhang, Fang Liu, Yanni Lou, Liqun Jia

**Affiliations:** 1 Graduate College, Beijing University of Chinese Medicine, Beijing, China; 2 Oncology Department of Integrative Medicine, China–Japan Friendship Hospital, Beijing, China; 3 Clinical Medical Research Institute, China–Japan Friendship Hospital, Beijing, China; 4 Pharmacy Department, China–Japan Friendship Hospital, Beijing, China

**Keywords:** cardiotoxicity, protocol, songjiao dihuang tang, statistical analysis plan, traditional Chinese medicine, tumour immunotherapy

## Abstract

**Background:**

While immune checkpoint inhibitors (ICIs) enhance tumour therapy, immune checkpoint inhibitor-associated myocarditis (ICIAM) pose risks of mortality because of their low incidence and rapid progression. Existing guidelines focus mostly on tiered management, with corticosteroid therapy serving as the primary treatment for the mild stage. Preliminary data suggest that Songjiao Dihuang Tang (SJDHT) may shorten disease duration, alleviate symptoms, and reduce glucocorticoid use. However, prospective multicentre studies remain rare because of sample scarcity, underdiagnosis of early-stage cases, centre-specific variability, and rapid clinical evolution. Consequently, in this study patients with mild ICIAM based on 2020 Chinese Expert Consensus criteria were recruited and it follows the 2023 clinical implementation recommendations. This prospective, multicentre RCT aims to verify whether SJDHT combined with conventional treatment reduces the disease duration, progression, and mortality.

**Methods and analysis:**

In this 1:1 randomized controlled trial, 200 patients with mild IC-IAM will receive either SJDHT (30 mL bid for 28 days) or a placebo. Both groups will receive standard care: initial methylprednisolone (1–2 mg/kg/d), tapered by 25%–40% every 3–5 days, and a switch to oral equivalent prednisone at ≤40 mg (tapered by 5 mg/week until cessation). A “stop&go” adaptive design is employed to manage rapid disease progression, with assessments at 24–72 h, day 14, and day 28 after enrolment. Weekly cTn monitoring informs MDT-guided steroid tapering (if cTn decreases) or potential unblinding and endpoint triggering (if the cTn levels increases with a confirmed progression risk). Following the 28-day treatment, follow-ups are scheduled at months 1, 3, and 5 (6 months total). Parameters such as cTn, myocardial enzymes, ECG, echocardiography, symptoms, MACE, and safety will be recorded at each visit. The primary outcomes are the cTn recovery time and treatment efficacy rate. Secondary outcomes include the ICIAM progression rate, cardiac function indices, cancer-related symptoms, quality of life, MACEs, and TCM efficacy. The data will be analysed according to a predefined statistical plan, including missing data imputation and primary/secondary/safety outcome evaluations.

**Discussion:**

This research will contribute a standardized Traditional Chinese Medicine clinical research paradigm to the global community, potentially improving the prognosis and safety of cancer patients receiving immunotherapy.

**Clinical Trial Registration:**

https://itmctr.ccebtcm.org.cn/. Trial number: ITMCTR2025001424. Registered on 16 July 2025 (retrospectively registered). Registry name:Therapeutic Strategies and Evidence-Based Research of Traditional Chinese Medicine (TCM) for Subclinical and Mild Immune Checkpoint Inhibitor-Associated Myocarditis (ICIAM).

## 1 Introduction

The clinical application of immune checkpoint inhibitors (ICIs) has significantly improved the prognosis of patients with various solid tumours; however, subsequent immune-related adverse events (irAEs) have emerged as critical factors that limit therapeutic efficacy and even threaten the lives of patients(Tan et al., 2022; Pathak et al., 2021; Le et al., 2025). Despite its low incidence (0.06%–3.8%), immune checkpoint inhibitor-associated myocarditis (ICIAM) is characterized by an extremely high mortality rate (39.7%–66%) and rapid disease progression (Wang et al., 2023; Li et al., 2025). Consequently, ICIAM has become among the most challenging and highly prioritized immune-related complications in clinical diagnosis and management. Currently, domestic research on ICIAM focuses primarily on exploring the molecular mechanisms, whereas international studies are predominantly retrospective (cohort or cross-sectional) in nature (Ostos-Mendoza et al., 2025). Although these studies contribute to elucidating the epidemiological characteristics, they remain limited in providing high-level evidence-based clinical recommendations. In the clinical management of ICIAM, international guidelines have evolved through numerous versions from the initial SITC to the NCCN (the NCCN guidelines have been updated 16 times), mainly recommending graded diagnosis and treatment, with glucocorticoids as the first-line choice for the mild stage and intensified therapy with immunosuppressants when steroids are ineffective. Given the unique advantages of traditional Chinese medicine (TCM) in preventing and treating the toxicity and side effects of radiotherapy and chemotherapy, our team, supported by the National Medical Center for Integrated Chinese and Western Medicine, has established an integrated Chinese and Western medicine diagnostic and therapeutic model. The preliminary exploration indicates that the application of Songjiao Dihuang Tang decoction (SJDHT) in this model for treating patients with mild ICIAM can significantly shorten the disease course, alleviate symptoms, and reduce the dosage of glucocorticoids. This model is expected to further improve the cure rate and reduce mortality and the rate of progression to severe disease. Therefore, in the present study, we intend to conduct a prospective clinical trial to verify the clinical value of the integrated Chinese and Western medicine model in reducing the mortality rate of ICIAM.

Currently, prospective studies (especially multicentre prospective studies) targeting ICIAM are extremely scarce (Feng et al., 2025), and the challenges are summarized in the following four points. First, due to the low incidence of ICIAM, single centres or regions with a low population density cannot easily meet the sample size requirements for clinical research. Second, mild cases—characterized primarily by elevated myocardial enzyme levels without symptoms or changes in cardiac structure—are easily missed in clinical practice, leading to the loss of therapeutic windows. Third, the management of this disease relies heavily on the diagnostic and therapeutic capabilities and the experience of multidisciplinary teams (MDTs) at the research units (Lehmann et al., 2023); variations in baseline treatment may lead to inconsistent efficacy across different centres. Fourth, given the rapid progression of the disease, a single-node continuous treatment protocol may pose ethical risks because of the inability to promptly detect disease progression and make appropriate clinical decisions.

A prospective, multicentre, double-blind, superiority randomized controlled trial has been designed and a “stop&go” adaptive model has been introduced in this study to address these issues. First, the collaborative participating units—China–Japan Friendship Hospital, Peking Union Medical College Hospital (CAMS), and Zhongshan Hospital affiliated with Fudan University—possess comprehensive MDTs and hold significant influence in the field of ICIAM in China, providing a robust guarantee for patient recruitment. Second, this study focuses on a population with mild ICIAM (those with mild symptoms, slight laboratory and ECG abnormalities, and no echocardiographic abnormalities), as initially proposed in the 2020 Chinese Expert Consensus on the Monitoring and Management of Immune Checkpoint Inhibitor-Related Myocarditis (Expert Committee on Cardio-Oncology of the Chinese Society of Clinical Oncology, 2020) and further refined in the 2023 Recommendations for the Clinical Implementation of Diagnosis and Treatment of Immune Checkpoint Inhibitor-Related Myocarditis (Wang et al., 2023). This study expands the traditional screening scope to increase the capture rate of the target population. Third, after referring to the 2020 Chinese expert consensus and relevant 2023 recommendations, this study has established a standardized and integrated clinical pathway aimed at minimizing heterogeneity in efficacy between centres. Finally, this study adopts the “stop&go” model, setting assessment nodes at 24–72 h after enrolment, during treatment, and at the end of treatment (days 14 and 28); subsequent diagnostic and therapeutic regimens will be dynamically adjusted based on the results of the assessment to ensure the timeliness and safety of treatment in patients. In summary, the design of this study balances scientific rigor with ethical considerations and possesses significant clinical research value.

## 2 Materials and methods

### 2.1 Study drug

#### 2.1.1 Preparation of SJDHT

SJDHT is composed of 5 Chinese botanical and animal-derived drugs, and the daily dosage of SJDHT decoction is as follows: 20 g of Shuiniujiao (*Bubalus bubalis* Linnaeus [Bovidae; Bubali Cornu]); 12 g of Mudanpi (*Paeonia suffruticosa* Andr [Paeoniaceae; Moutan cortex]); 30 g of Shengdihuang (*Rehmannia glutinosa* Libosch [Orobanchaceae; Radix rehmanniae]); 30 g of Yousongjie (*Pinus tabuliformis* Carr [Pinaceae; Pini lignum nodi]); and 12 g of Baishao (*Paeonia lactiflora* Pall [Paeoniaceae; Paeoniae radix alba]). The components (standardized, sliced raw materials) of the TCM decoction used in this study—none of which contained controlled substances or materials derived from endangered species—were procured from Sinopharm Group Beijing Huamiao Pharmaceutical Co., Ltd (Beijing, China). All the botanical and animal-derived drugs were verified by the manufacturer to comply with the standards of the Chinese Pharmacopoeia (ChP, 2020 Edition, Volume I), as detailed in Supplementary Material S1. For the preparation of SJDHT, the botanical and animal-derived drugs—Rehmanniae Radix, Pini Lignum Nodi, Paeoniae Radix Alba, Moutan Cortex, and Bubali Cornu—were mixed at a dry weight ratio of 15:15:6:6:10. The mixture was subjected to two cycles of aqueous extraction, first with 10 volumes of distilled water (w/v) and then with eight volumes, for 1 h in each cycle. The resulting filtrates were pooled and concentrated under reduced pressure to achieve a final crude drug concentration of 0.67 g/mL (corresponding to a raw material-to-liquid ratio of 1:1.5). The identification and characterization of SJDHT decoction followed the procedures outlined in *The ConPhyMP—Guidelines* (Heinrich et al., 2022), with the full checklist provided in Supplementary Material S2.

### 2.2 SJDHT-medicated serum preparation

Six male SD rats (180 ± 20 g) were selected for this study. These rats were then divided into two groups with three rats in each group: an SJDHT group and a control group. Each rat was gavaged with 0.9% normal saline or the SJDHT solution (1.73 g/mL of drug) at a dosage of 2 mL/rat twice a day for seven consecutive days. Two hours after the final administration, the rats were anaesthetised with sodium pentobarbital. Blood was subsequently collected from the abdominal aorta and centrifuged to isolate serum. The serum was heat-inactivated in a 56 °C water bath for 30 min, sterilized by filtration through a 0.22 μm needle filter, and stored at −80 °C for further use. This technical service was provided by Youke Zhuoyue Biomedical Technology Co., Ltd (Beijing, China).

### 2.3 UPLC‒MS analysis of SJDHT

The chemical composition of the aqueous extract was determined by the Pharmaceutical Technology Core Facility (School of Pharmaceutical Sciences, Tsinghua University) using ultrahigh-performance liquid chromatography‒mass spectrometry (UPLC‒MS) analysis. The column was an ACQUITY UPLC HSS T3 (100 mm × 2.1 mm, 1.8 μm) column with a column temperature of 35 °C. Mobile phase A was 0.1% formic acid-water, mobile phase B was 0.1% formic acid–acetonitrile, and the flow rate was 0.25 mL/min. The data were acquired using a Waters Synapt G2-Si QTOF mass spectrometer. The analysis was performed strictly according to the set elution gradient. In accordance with the set mass spectrometry parameters, the mass spectrometry signal of the sample was collected in both positive ion and negative ion scanning modes. The methods and results are available in Supplementary Material S3).

### 2.4 Management of gastrointestinal adverse events

Based on clinical experience, diarrhoea is the primary adverse event associated with SJDHT. According to the study protocol, the composition and dosage of SJDHT will remain unaltered upon the onset of diarrhoea. However, symptomatic treatment with loperamide hydrochloride capsules (Imodium) should be proactively administered to manage this adverse reaction.

## 3 Study design

**FIGURE 1:**
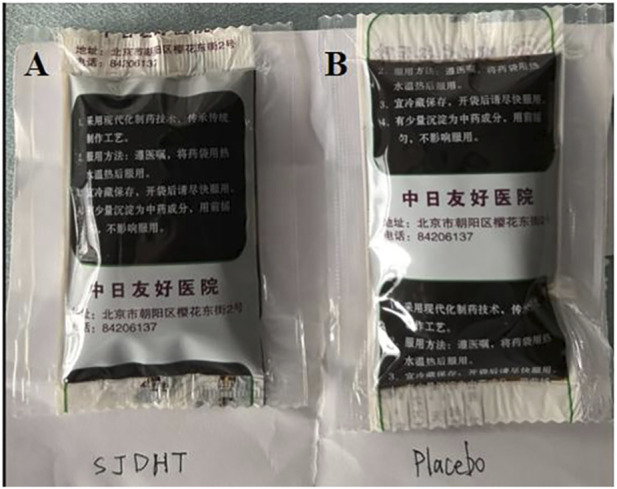
SJDHT decoction and placebo (A) Appearance and packaging of the SJDHT decoction (B) appearance and packaging of the placebo.

**FIGURE 2:**
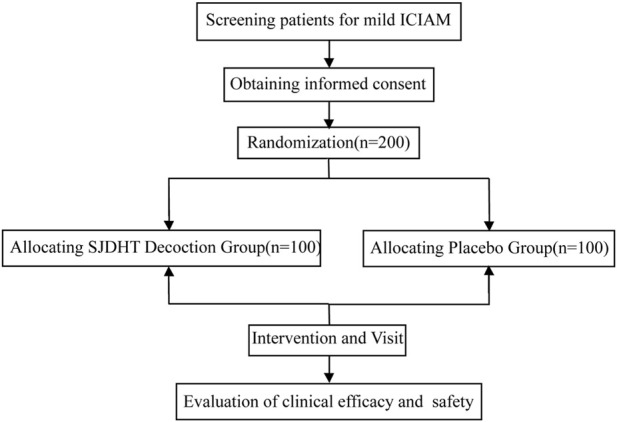
Flow diagram of this study.

A total of 200 patients diagnosed with mild ICIAM will be enrolled in this study. The patients will be divided into two randomized groups and treated with either SJDHT decoction or the placebo. This protocol adheres to the SPIRIT guidelines of the interventional trials reported by Chan et al., in 2013 Supplementary Material S4. The true drugs and the placebo were manufactured by China–Japan Friendship Hospital (Figure 1). The study procedure is summarized in Figure 2. The details of the schedule for enrolment, interventions, and assessments in this study are presented in Table 1.

**TABLE 1:** SPIRIT schedule of enrolment, interventions and assessments Visit project Study period.

Schedule of assessments	Screen period (baseline)	Allocation	Visit (0 + n days)							
			Initial assessment	Trial period				Other visits		Viability visits
Time of visit (day)	-7–−1	0 (first day)	3 ± 1	7 ± 3	14 ± 3	21 ± 3	28 ± 3	56 ± 3	112 ± 7	Q24 W (±14)
1. Enrolment										
Informed consent	​	√	​	​	​	​	​	​	​	​
Inclusion/exclusion criteria	√		​	​	​	​	​	​	​	​
Demographic data	√		​	​	​	​	​	​	​	​
Medical history	√		​	​	​	​	​	​	​	​
Vital signs	√		​	​	​	​	​	​	​	​
Physical examination	√		​	​	​	​	​	​	​	​
Random allocation	​	√	​	​	​	​	​	​	​	​
2. Interventions										
Glucocorticoids (GCs)	​	√	√	√	√	√	√	…	​	​
Administration of study drugs	​	√	√	√	√	√	√	​	​	​
3. Assessments										
Cardiac biomarker panel T (four items)	√		√	√	√	√	√	√	√	​
cTnI level	√		√	√	√	√	√	√	√	​
Cardiac enzyme profile	√		√	√	√	√	√	√	√	​
Markers of liver and renal function	√		​	​	​	​	√	√	√	​
Routine blood analysis + high-sensitivity CRP levels	√		​	​	​	​	√	√	√	​
ESR	√		​	​	​	​	√	√	√	​
Cytokine profiling	√		​	​	​	​	√	√	√	​
Lymphocyte subsets	√		​	​	​	​	√	√	√	​
Coagulation (six items)	√		​	​	​	​	√	√	√	​
Routine urinalysis	√		​	​	​	​	√	√	√	​
Stool tests	√		​	​	​	​	√	√	√	​
Cardiac ultrasound	√		​	​	​	​	√	√	√	​
12-Lead ECG	√		​	​	​	​	√	√	√	​
4. Assessment of the current status										
Symptoms	√		√	√	√	√	√	√	√	​
Tongue and pulse	√		√	√	√	√	√	√	√	​
KPS score	√		​	​	​	​	√	√	√	​
MDASI-TCM scale	√		​	​	​	​	√	√	√	​
TCM syndrome score	√		​	​	​	​	√	√	√	​
Adverse event monitoring	​	​	√	√	√	√	√	√	√	​
Concomitant medication	√		√	√	√	√	√	√	√	​
Glucocorticoid therapy	√		√	√	√	√	√	√	√	​
Tumour assessment	​	​	​	​	​	​	​	​	√	√
Anti-tumour therapy	​	​	​	​	​	​	√	√	√	√
MACEs	​	​	​	​	​	​	√	√	√	√
5. Other tasks										
Distribute drugs (14 days)	​	√	​	​	√	​	​	​	​	​
Recycle residual drugs	​	​	​	​	√	​	√	​	​	​
Blood sample collection	√ (selective sampling)	​	​	​	​	​	√ (correspondence to the previous test)	​	​	​
Stopandgo	​	​	√	​	√	​	√	​	​	​
Viability assessment	​	​	​	​	​	​	​	​	​	√

### 4 Inclusion criteria

### 4.1 diagnostic criteria for mild ICIAM

The diagnostic criteria for mild ICIAM are based on the Clinical Practice Recommendations for the Diagnosis and Treatment of Immune Checkpoint Inhibitor-Associated Myocarditis (2023) and the Guidelines for the Management of Toxicity Associated with Immune Checkpoint Inhibitors (2021), and individuals who qualified for eligibility and met the following criteria will be included.

1. Routine activities can elicit nonspecific mild symptoms, including fatigue and dyspnoea.
2. The levels of myocardial injury markers (cTn, CK, CK-MB, and AST) and natriuretic peptides are mildly elevated.
3. Mild ECG abnormalities, including new-onset sinus tachycardia, atrial arrhythmias, and nonspecific ST-T changes, are observed.
4. No abnormalities in cardiac structure or function are detected on echocardiography or cardiac MRI.
5. Provided written informed consent before enrolling in the trial.

### 4.2 TCM diagnostic criteria for mild ICIAM(heat-consuming yin deficiency)

The diagnostic criteria for mild ICIAM are based on the TCM Clinical Diagnosis and Treatment Terminology Part 2: Syndromes (GB/T 16,751.2–2021), and individuals who qualified for eligibility by meeting the following criteria will be included (the presence of two primary symptoms plus one secondary symptom, with reference to the tongue and pulse diagnosis).

1. Primary symptoms of ① fever or “five-centre heat” sensation, ② thirst and desire to drink, ③ excessive sweating or night sweats, and ④ dry skin.
2. Secondary symptoms of ① trouble sleeping or feeling mentally agitated; ② bodily emaciation; ③ oliguria with dark yellow urine or constipation with hard stools; and ④ haematemesis, haemoptysis or epistaxis.
3. Tongue and pulse diagnosis including a thin and small tongue with a red or crimson tongue body, possibly with cracks or papillae, with little or no fur and a fine or rapid pulse.

### 4.3 Therefore, patients are eligible for the study if they meet all of the following criteria

1. Confirmation of malignancy through a cytological or histopathological diagnosis.
2. Developed ICIAM following treatment with immune checkpoint inhibitors (ICIs), which meets ①.
3. TCM syndrome differentiation is performed by two licenced TCM practitioners (kappa≥0.8) and conforms to ②.
4. KPS score >60, expected survival >6 months and 18 ≤ age ≤80 years.

## 5 Exclusion criteria

Patients are ineligible for this study if they meet any of the following criteria.

1. Some definitive evidence indicating that cardiac injury is not attributable to ICIs.
2. Severe immune-related adverse events (irAEs) involving organs other than the heart.
3. Received glucocorticoid therapy for more than 1 week.
4. Patients who have significant cardiac, pulmonary, hepatic, or renal dysfunction or severe comorbidities such as heart failure, cerebral infarction, and chronic obstructive pulmonary disease (COPD).
5. Patients with known hypersensitivity or contraindications to any study-related medications, any botanical and animal-derived drugs of the SJDHT decoction, or any of its excipients.

## 6 Withdrawal criteria and termination criteria

### 6.1 Withdrawal criteria

1. Researchers will remove patients from a trial for illnesses that make further study unsuitable, adverse reactions that preclude continuing, or any health risks from ongoing participation.
2. Patients have the right to leave a trial at any time. Withdrawals are noted if patients stop treatment or are lost to follow-up, with reasons documented precisely.
3. The principal investigator and statisticians decide whether a patient should be excluded before analysis, considering study progress and reasons for withdrawal, especially if participants fail to meet the inclusion criteria or lack postrandomization data.
4. Patients will be withdrawn from the study or the protocol will be terminated if any of the following criteria are met, regardless of cardiac troponin (cTn) levels.
5. Objective Clinical Deterioration: Occurrence of acute hemodynamic instability, refractory dyspnea (NYHA IV or worsening of ≥1 grade), or new-onset life-threatening rhythm disturbances (e.g., sustained ventricular tachycardia, high-degree AV block).
6. Echocardiographic Decline: A significant reduction in left ventricular ejection fraction (LVEF), defined as an absolute decrease of $>10\% from baseline or a decline to an absolute value below $35\%$.
7. MDT Triggered Intervention: Any clinical scenario where the Multidisciplinary Team (MDT)—comprising senior cardiologists, intensivists, and the principal investigator—determines that continued adherence to the study protocol poses an unacceptable risk to the patient.

In such instances, the MDT will be convened immediately to evaluate the benefit-risk ratio. The team reserves the right to initiate emergency rescue medication or mandate study withdrawal to prioritize standard-of-care treatments and patient safety.

### 6.2 Termination criteria

A trial may be stopped early because of a high rate of serious adverse events, significant protocol errors, ethics committee requests, or administrative cancellations.

## 7 Intervention measures

### 7.1 Screening period

**FIGURE 3:**
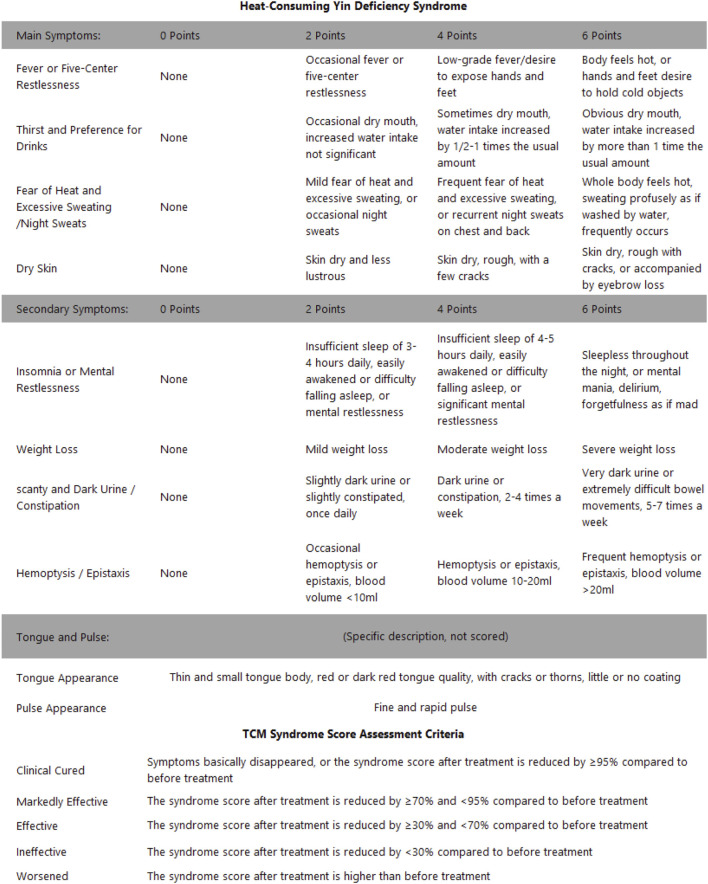
TCM symptom scoring form.

A comprehensive physical examination will be completed within 7 days prior to the initiation of the study medication; blood will be collected from each subject measure cardiac biomarker panel T (four items), troponin I (cTnI) levels, the cardiac enzyme profile, liver and renal function markers, routine blood counts and high-sensitivity C-reactive protein (CRP) levels, cytokine profiles, lymphocyte subsets, coagulation (six items), routine urinalysis parameters, stool tests, cardiac ultrasound and 12-lead ECG examinations. The medical history, quality of life questionnaires (MDASI-TCM scale), and TCM symptom scoring forms will be completed (Figure 3).

### 7.2 Trial period

**FIGURE 4:**
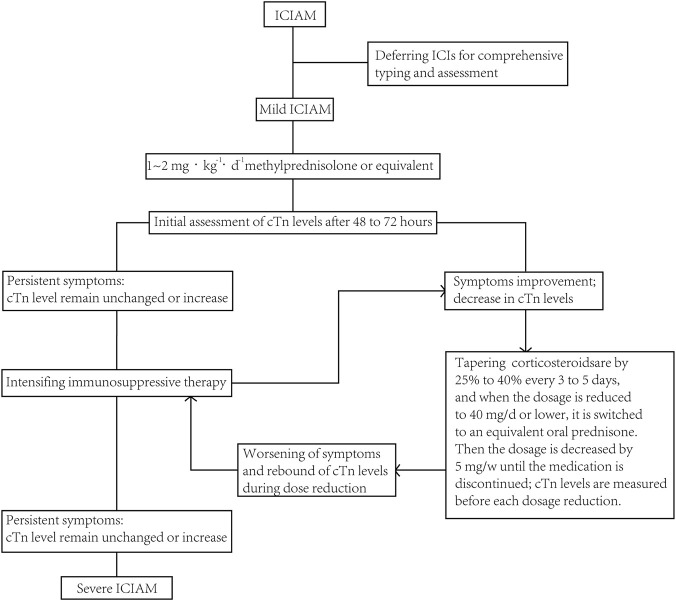
Pathway for the diagnosis and treatment of mild ICIAM. Note: Severe ICIAM is categorized by: obvious symptoms (e.g., fatigue, palpitations, chest pain, myalgia) during daily activities, but without haemodynamic changes; and/or significantly elevated biomarkers and natriuretic peptides; and/or new ECG findings (e.g., Grade I–II AV block, bundle branch block, intraventricular conduction block, frequent PVCs, QT prolongation, diffuse ST-segment elevation, T-wave changes, or decreased R-wave amplitude/abnormal Q-waves, excluding acute MI); and/or structural/functional abnormalities on echocardiography or CMR.

Patients in the SJDHT group will receive the SJDHT decoction (30 mL per bag), one bag at a time, two times a day, to be swallowed with warm water. The administration of the placebo decoction (30 mL per bag) will be the same as that in the SJDHT group (with the option to use glucocorticoids at a dosage of 1–2 mg kg^-1^·d^-1^ [methylprednisolone] according to medical practice and clinical judgement) (Figure 4). The treatment period will commence on the day of enrolment and continue for a total of 4 weeks, encompassing the initial assessment (two to three days after the initial dosing), the second assessment (14 days after the initial dosing), and the third assessment (28 days after the initial dosing). These three assessments will involve a re-evaluation of the patient’s clinical condition and an appraisal of the status of protocol implementation. Based on the findings of this evaluation, subsequent diagnostic and therapeutic regimens will be dynamically adjusted to ensure the prompt diagnosis of disease progression and the ethical safety of the research. The medication can be stopped in advance if the discharge standard is reached. Subsequently, follow-up will be performed to monitor disease progression and outcomes.

#### 7.3.1 Basic Treatment and Follow-up

Standard treatment follows the therapeutic pathway with methylprednisolone administration. Participants are observed and followed until their cardiac troponin (cTn) level returns to baseline. An initial follow-up with an increase in the cTn level of more than 50% will trigger study endpoints, unblinding, and withdrawal from the study group. Participants will then be administered increased doses of steroids and other intensive medical treatments, such as Janus kinase (JAK) inhibitors. After 48–72 h of treatment, participants will be re-evaluated. If symptoms worsen or cTn levels increase again by more than 50%, the patient will be promptly provided with intensive care treatment.

#### 7.3.2 Concomitant medications and treatment

1. Permitted: Symptomatic supportive treatment in accordance with guidelines and clinical symptoms; continuation of medications or other therapeutic methods required for other diseases.
2. Prohibited: Other TCMs related to the treatment of ICIAM (including botanical and animal-derived drugs with the effects of invigorating qi, nourishing yin, clearing heat, detoxifying, etc.), which encompass decoctions, decoction, proprietary Chinese medicines, and therapeutic practices such as acupuncture, gua sha, and blood-letting therapy.

The types, dosages, and cumulative times (including the start and end dates) of the combined therapeutic drugs should be recorded during the observation period. The intervention will be stopped if any severe adverse effects occur or the patient withdraws.

#### 7.3.3 Visits during the trial period

1. Medication Period Visits (Regular Assessments, Weeks 1, 2, three and 4): Weekly follow-ups will be conducted through voluntary hospital visits by the patients to assess their general health status, during which the cardiac enzyme profile, cTnI levels and cardiac biomarker panel T (four items) will be assessed. At week 4, comprehensive physical examinations and other examinations (consistent with those in the screening period) will be performed. Routine urinalysis, stool tests, and assessments of liver and renal function will be performed for safety evaluation. Prospective monitoring and documentation of major adverse cardiovascular events (MACE) will be performed throughout treatment. In this study, MACEs are defined as a composite of cardiovascular mortality, cardiac arrest, cardiogenic shock, and advanced atrioventricular block. (Herrmann et al., 2025).
2. Other Visits (Weeks 8 and 16): The assessments will be voluntary hospital visits to evaluate the general health status, during which participants will complete examinations (consistent with those in the screening period) to assess long-term efficacy and the recovery status. Prospective monitoring and documentation of MACEs will be performed.
3. Safety Assessment: This assessment will be conducted in the examinations (consistent with that in the screening period); if safety is confirmed, no further assessments will be performed. Conversely, the assessments will continue until toxicity resolves, a return to baseline levels is observed, or irreversible effects occur.
4. Viability Assessment: Survival follow-up visits will be scheduled at 168-day (±14 days) intervals after the first day of the trial period until the participant’s death, loss to follow-up, study discontinuation, or other criteria for study completion are met.
5. Unscheduled Visits: As deemed necessary by the investigators, unscheduled visits may be conducted to collect additional clinical and laboratory data. These visits aim to understand overall survival (OS), progression-free survival (PFS), and the rate of rechallenge with ICIs and to explore peripheral blood mononuclear cell sequencing to obtain deeper insights into the mechanisms of action.

Note: During follow-up, the glucocorticoid dose will be gradually tapered based on clinical assessments. The administration of the SJDHT decoction or placebo decoction is an intervention specific to this study, and these treatments will be provided at no cost by the research institute.

## 8 Outcome measures

### 8.1 Primary outcomes

cTn Recovery Time: The time from enrolment to the normalization of cTn levels (the number of days).

### 8.2 Secondary outcomes

#### 8.2.2 Treatment Efficacy Rate

It is calculated as (Number of Cured + Markedly Effective + Effective)/Total Number of Patients × 100%. A cure is defined as the disappearance of symptoms and normalization of cTn levels; markedly effective refers to the near disappearance of symptoms with a ≥50% decrease in cTn levels; effective indicates either a 30% to <50% decrease in cTn levels regardless of symptom changes or a marked improvement in symptoms with a <30% decrease in cTn levels; and ineffective is characterized by no improvement in symptoms, a <30% decrease in cTn levels, or an increase in cTn levels.

#### 8.2.3 ICIAM Progression Rate

This parameter is defined as the number of patients who transition from mild to severe symptoms divided by the total number of patients with mild symptoms at enrolment, multiplied by 100%.

#### 8.2.4 Cardiac Function and Related Indicators.

Cardiac injury markers (cTnI, cTnT, and CK-MB), natriuretic peptides, D-dimer, AST, inflammatory markers (ESR, CRP, and cytokines), electrocardiogram, and echocardiography will be assessed.

#### 8.2.4 Tumour-Related Symptoms and Quality of Life Scoring

MDASI-TCM questionnaire.

#### 8.2.5 TCM Syndrome Efficacy Rate.

It is calculated as (Number of Recovered + Markedly Effective + Effective)/Total Number of Patients × 100%.

#### 8.2.6 ${MACE}_{s}$

The occurrence of MACEs during the follow-up period will be assessed.

### 8.3 Exploratory outcomes

Assessments of overall survival (OS), progression-free survival (PFS), the rechallenge rate of ICIs and peripheral blood single-cell sequencing will be performed to explore in depth the efficacy mechanisms.

### 8.4 Safety outcomes and adverse events (AEs)

AEs will be continuously checked during the treatment period. AE indices will include all adverse events. The AEs will be graded as mild, moderate, or severe. Mild AEs could be defined as situations that could be tolerated without any further medical therapies. Moderate AEs are pain or discomfort that patients cannot tolerate and require medical treatment. Severe AEs indicate that, after receiving the medications used in this study, the patients die; experience life-threatening, permanent, or severe disability or loss of function; need hospitalization or extended hospitalization; or congenital abnormalities or birth defects occur in their children (Dy et al., 2018).

#### 8.2.7 AE Documentation and Reporting Pathways

In the safety analysis, all participants within the safety set will be monitored. AEs occurring during the intervention must be meticulously documented in medical records or laboratory reports, detailing the time of occurrence, clinical manifestations, severity, duration, frequency, management, outcome, relationship to the intervention medication, and any serious AEs leading to the discontinuation of the study drug. Subsequently, the number and frequency of AEs in both groups will be calculated. In cases of serious AEs, a serious adverse event report form must be completed and sent to the research centre, principal sponsor, and research medical ethics committee as soon as possible. Moreover, the National Medicinal Product Administration will be notified within 24 h. Other AEs must also be documented as soon as possible.

#### 8.2.8 Management of AEs

Monitoring AEs: The follow-up of each adverse reaction should continue until the issue is resolved, returns to baseline levels, is confirmed as unresolvable/permanent, or results in death.

Serious AEs: Investigators and other responsible parties should clinically analyse and determine the causal relationship between serious AEs experienced by subjects and the trial treatment regimen.

## 9 Sample sizes

The hypothesis of this study is that the efficacy of SJDHT is significantly superior to that of the placebo. The median time to alleviation will be compared between the two groups using the log-rank test (Lakatos method). Clinical experience and the literature suggest that the median time to alleviation for the control group is 151 days, whereas for the experimental group, it is 68 days(Zhang et al., 2021). The study duration is set at 4 years, with a recruitment period of 3 years. For superiority testing (statistical superiority), a one-sided α of 0.025 and β of 0.2 are selected, with a ratio of the experimental group to the control group of 1:1. Utilizing PASS 11.0 software, 88 patients are required for each group. Accounting for a dropout rate of 10% and the length of the study period, 100 patients in each group are needed, totalling 200 patients.

## 10 Recruitment

A multifaceted recruitment strategy will be implemented to ensure that the target sample size is achieved within the projected timeframe. (1) Research assistants will routinely screen electronic medical records and oncology outpatient logs to identify potential candidates. Recruitment posters and brochures will be displayed in outpatient waiting halls and oncology inpatient departments at the China–Japan Friendship Hospital and its satellite centres. These posters and brochures will describe the study objectives, drugs, medical examinations, and study eligibility conditions and application method. (2) Standardized recruitment announcements, approved by the Institutional Review Board (IRB), will be disseminated via official WeChat accounts to increase visibility among the general public and patient communities. (3) A collaborative referral mechanism will be established with affiliated medical institutions to facilitate the transfer of patients meeting the predefined inclusion criteria.

The recruitment process started in December 2024 and will last until July 2028. Patients will not participate in the study design, recruitment, research procedures, analysis, or dissemination of findings. Recruitment progress will be monitored monthly by the Trial Steering Committee to assess enrolment rates and implement corrective actions if necessary.

## 11 Randomization and allocation

Beijing Yinreida Medical Technology Ltd. Will be responsible for randomization. The randomization method will be employed to allocate a sample size of 200 participants into a 1:1 ratio between the experimental group and the control group using SAS statistical software and the Interactive Web Response System (IWRS). Participants will be competitively enrolled in this study. The randomization code will be concealed in opaque envelopes, and the allocation information will not be known before the randomization procedure.

## 12 Blinding

All the medications used in this study will be administered under good manufacturing practices. The research assistants who allocate patients to groups will be unaware of their treatment allocation before grouping. Before the analysis, evaluation or unblinding, all the samples and data will be anonymized to the researchers, assessors and statisticians. The specific details of the implementation are as follows.

### 12.1 placebo development

The placebo is manufactured by the Pharmaceutical Manufacturing Division, Department of Pharmacy, China–Japan Friendship Hospital, to match the experimental SJDHT decoction in colour, odour, taste, shape, and texture, comprising 10% SJDHT decoction and 90% other excipients. to ensure indistinguishability by both the clinical trial researchers and participants (Table 2). Previous studies have demonstrated that the preparation method—utilizing diluted raw drugs supplemented with excipients—is pharmacologically inert and maintains high similarity to the decoction(Zhang et al., 2015; Tang et al., 2009). The specific preparation involves fully dissolving Astragalus gum in 40 °C water under constant agitation, followed by the sequential addition of the remaining components.

**TABLE 2:** Comparison between the SJDHT decoction and placebo.

No.	Item	Characteristics of the SJDHT decoction	Placebo components
1	Colour and lustre	Dark black	1.5‰ caramel pigment
2	Texture	Slightly viscous and homogeneous	0.8‰ astragalus gum
3	Basic flavour	Sweet	0.2‰ polydextrose
4	Basic flavour	Bitter	0.09‰ bittering agent
5	Basic flavour	Sour	1‰ food-grade citric acid
6	TCM characteristics	TCM characteristics	10% genuine medicine

### 12.2 Medication packaging

The placebo and the active treatment are identical in dosage form, appearance, packaging, labelling, and identification, with clear indications for exclusive use in clinical trials.

### 12.3 Packaging and labelling

Individuals uninvolved in the clinical observations, monitoring, and statistical analysis of participants in this trial will encode the trial medication and placebo according to a pre-established randomization sequence. The corresponding drug numbers will be prominently displayed on the exterior packaging.

### 12.4 Emergency envelope preparation

1. The envelopes will be sealed and opaque and imprinted with emergency letters for the SJDHT decoction clinical trial, drug numbers, and protocols for emergency unblinding.
2. The envelopes contain the medication information and treatment procedures for the subject. These envelopes are distributed with the trial medication to each centre and collected uniformly after the conclusion of this trial.

### 12.5 Blinding record keeping

The encoding process is documented by the encoder, known as the blinding record, which is retained as part of the documentation of this clinical trial. It includes details such as drug packaging, administration instructions, storage requirements, drug distribution methods, generation of the random sequence, packaging for each subject, emergency letters, storage of the blinding key, and protocols for unblinding.

### 12.6 Storage of the blinding key

The random sequence, along with parameters such as the random seed number and block length used to generate the random numbers, is sealed in triplicate and preserved by the principal investigator, the China–Japan Friendship Hospital Science and Technology Center, and a third-party data management organization. No individual is permitted to privately retain the blinding key or emergency letters. Unauthorized access to the blinding key is considered a failure of the clinical trial, and unauthorized access to emergency letters is treated as a dropout case.

### 12.7 Unblinding

In cases of necessity, either when medical treatment of the subject requires knowledge of the study medication or when a medical emergency necessitates immediate identification of the medication taken by the patient, emergency unblinding should occur under the following conditions.

1. Emergency Unblinding—Before unblinding, attempts should be made to notify the researchers and relevant personnel at the China–Japan Friendship Hospital Science and Technology Center. If contact with the researchers cannot be established prior to unblinding, they must be contacted within 24 h after unblinding. The researchers are required to document the time, location, and reason for unblinding.
2. Unblinding Protocol—This trial plans to use a single unblinding method, which will occur after the database is locked, to identify the treatment plans of the two groups.

### 12.8 Evaluation of placebo blinding efficacy

Comparison of Characteristics Between the Placebo and SJDHT Decoction: Appropriate amounts of the placebo and SJDHT were placed in two transparent plastic cups. Trial participants were informed that the two were different while being ensured to remain unaware of the specific positions of the placebo and the active drug. Multiple trial participants (≥10) were asked to evaluate the degree of difference between them in terms of colour, smell, touch, taste (bitterness, sourness, sweetness, and medicinal flavour), and overall perception.

Ability to Identify the Formulations: Fourteen transparent plastic cups, with seven containing SJDHT and the remaining seven containing the placebo, were prepared. Using a syringe ensures that each cup contains equal amounts of the respective preparation. Multiple trial participants (≥10) were allowed to freely select cups for tasting, and they were asked whether they believed the cup contained SJDHT or the placebo (Table 3).

**TABLE 3:** Form for the evaluation of placebo blinding efficacy.

Item	Conclusions	​	​	​	​
​	Fully consistent	Relatively consistent	Ambiguous	Relatively inconsistent	Fully inconsistent
Colour	​	​	​	​	​
Smell	​	​	​	​	​
Touch	​	​	​	​	​
Bitterness	​	​	​	​	​
Sourness	​	​	​	​	​
Sweetness	​	​	​	​	​
Medicinal Flavour	​	​	​	​	​
Overall Perception	​	​	​	​	​

​	Correct	Ambiguous	Incorrect	​	​
SJDHT	​	​	​	​	​
Placebo	​	​	​	​	​

## 13 Adherence to the study protocol

During treatment, compliance with the protocol will be evaluated at every follow-up, with a focus on how well patients take their medications. The proportion of participants in each group who adhere to the medication regimen will be assessed through frequency statistics. Adherence to medication between the two groups will be assessed by comparing the ratio of the dosage actually administered to patients with the dosage that was prescribed. Daily records will be kept for all medications that are either provided to patients or not administered.

## 14 Data collection, management, and quality control

### 14.1 Data collection, management and confidentiality

Participant data, including demographics, anthropometric measurements, medical history, and prior treatments, will be documented using both paper and electronic case report forms (eCRFs). Data management will be facilitated via a secure, web-based electronic data capture (EDC) system developed by Beijing Yinruida Medical Technology Co., Ltd. Researchers (including investigators and research assistants) at each clinical site are responsible for chronological data entry into the eCRFs. The coordination team from China–Japan Friendship Hospital will provide standardized EDC training and ongoing technical support to ensure data integrity and security. To enhance participant retention and ensure comprehensive follow-up, Researchers will periodically notify patients via telephone and WeChat regarding their scheduled hospital follow-up appointments.

In this trial, the eCRF will align with details from the original medical records, laboratory reports, and other relevant sources. In accordance with the study protocol, the eCRF is designed to ensure that all data necessary for the analysis are gathered in an efficient and accurate manner. The sponsor and investigators will implement quality control and assurance measures to maintain data quality and ensure compliance with the protocol. Researchers involved in clinical research at each clinical site must possess the requisite expertise, qualifications, and skills in clinical research. They must clearly articulate the protocols regarding patient recruitment, data entry, medication administration, adverse event documentation, and dropout records. They should also diligently and patiently educate patients to ensure full understanding and cooperation, thereby improving patient compliance. Patients must be made aware of the potential side effects of the medication and the strategies to be employed in the event of such occurrences.

The authenticity, integrity, and confidentiality of clinical research data must be safeguarded, and the traceability of data must be ensured. Any discrepancies in the data will be input by the data manager into the data readiness queue (DRQ) and dispatch a query to the investigators via the clinical monitor. The investigators are expected to promptly respond to these queries. The data manager will then revise the data based on the investigators’ feedback and verify and re-enter them, potentially resubmitting the DRQ if further adjustments are needed. Data verification encompasses automated program checks, manual checks, and verification meetings. Any identified inconsistencies must be promptly rectified. The data department is responsible for generating a discrepancy report for the researchers’ confirmation and subsequent amendments. Upon study completion, researchers and statisticians will review and lock the data once they are confirmed to be accurate. All electronic data will be securely stored in password-protected files on designated computers accessible to the researchers. The necessary documentation for this study will be archived and managed by the relevant drug clinical research facilities and statistical departments. The results of this project may be published in medical journals, but we will maintain the confidentiality of the patients’ information in accordance with legal requirements. Unless required by the relevant laws, patients’ personal information will not be disclosed. When necessary, government regulatory departments and hospital ethics committees and their personnel can review patients’ records as stipulated. The research data will be retained for 5 years following the conclusion of this study.

### 14.2 Quality control

#### 14.2.1 Uniform Basic Treatment Protocol

In 2023, “Clinical Diagnostic and Treatment Implementation Suggestions for Myocarditis Related to ICIs”, led by Fudan Zhongshan Hospital, first proposed a consensus on specific diagnostic and treatment pathways. This consensus will serve as the foundation for the integration of disease and syndrome study design and the formulation of a basic treatment plan, ensuring the basic safety of treatment for the mild stage of severe oncological heart disease and other stages. Each centre will refer to the standard basic treatment plan to minimize various biases associated with multicentre research.

#### 14.2.2 Resource Accessibility, Technical Expertise, and Robust Infrastructure.

The materials required for this study are fully accessible. Voucher samples of the components of the decoction, including Rehmanniae Radix, Bubali Cornu, Paeoniae Radix Alba, Moutan Cortex, and Pini Lignum Nodi (Voucher No. 000675), as well as the SJDHT decoction and its matching placebo (Voucher No. 000676) have been deposited in the cold storage of the Pharmaceutical Preparation Room at China–Japan Friendship Hospital for future reference and verification.

This study has a team with complete technology: the leading unit of the project, the China–Japan Friendship Hospital team, has established a database of 263 cases of irAE “disease–syndrome–phenotypes”, including 29 patients with ICIAM. Based on the preliminary clinical syndrome differentiation, the pathogenesis of ICIAM has been shown to transition from the initial stage to toxic damage to the heart/qi and yin deficiency syndrome (subclinical stage) to heat burning yin injury syndrome (mild stage), guiding the establishment of basic treatment principles for mild ICIAM. Moreover, for nearly 30 years, this team has been dedicated to the study of TCM for the prevention and treatment of AEs in tumour treatment and is a leading team in this field at home and abroad, having won 8 national and provincial-level scientific and technological progress awards. Relying on the China Association of TCM, it has taken the lead in issuing six related guidelines or consensuses. Based on the National Integrated Traditional Chinese and Western Medicine Center, it has established an integrated traditional Chinese and Western medicine multidisciplinary MDT for irAEs, treating more than a thousand patients with irAEs annually. It has established a multicentre clinical database for irAEs and published the first domestic study on the syndrome of irAEs (Therefore, this study will include 100 patients from China–Japan Friendship Hospital and 100 patients from other centres, with the goal of reducing bias.) The technical team and the preliminary foundation of the project are complete.

### 14.3 Data monitoring and auditing

An independent Data Monitoring Committee (DMC), comprising clinical and methodological experts from China-Japan Friendship Hospital, will oversee participant safety and data validity in accordance with a predefined charter; the DMC maintains full independence from the sponsor with no competing interests. A formal interim analysis for efficacy and safety is scheduled upon 50% recruitment using the O’Brien-Fleming group sequential method, with the DMC holding the authority to recommend trial termination to the Steering Committee based on predefined stopping boundaries. Furthermore, independent audits of trial conduct and regulatory compliance will be performed annually, separate from the investigators and sponsor, to ensure rigorous adherence to the protocol and Good Clinical Practice (GCP) guidelines.

### 14.4 Biospecimen collection and management

Biological specimens for molecular analysis will be collected and stored in strict adherence to institutional standardized operating procedures to ensure longitudinal sample integrity. Residual materials will be archived in a regulated biorepository for potential ancillary research, managed under a framework of rigorous ethical compliance and informed consent.

### 15 Statistical analysis plan

The general goal is to use the internationally recognized SPSS 25.0 statistical software package for computations. Unless specified otherwise, all statistical significance tests in this trial will be conducted using two-tailed tests, with a P value of ≤0.05 serving as the criterion for determining a statistically significant difference.

### 15.1 Definition of analysis sets

#### 15.1.1 Full analysis set (FAS)

The entire dataset will be subjected to an intention-to-treat analysis, which includes all individuals who were randomly allocated to a group, who took the medication at least once, and who attended at least one visit.

#### 15.1.2 Per protocol set (PPS)

This set encompasses participants who adhere to the guidelines of this study and maintain complete data for crucial initial metrics and the main outcome measure. Those with significant deviations in concurrent medication use or a markedly low level of adherence will typically be disqualified from the PPS.

#### 15.1.2 Safety set (SS)

Inclusion will be granted to any randomized subject who has been treated with at least a single dose of the medication and has undergone a minimum of one safety evaluation. For the SS, no statistical imputation will be applied to the safety data.

### 15.2 Descriptive statistics

The analysis will utilize the intention-to-treat (ITT) dataset. Descriptive statistics will be employed to summarize demographic data and other baseline characteristics and to compare group equilibrium: continuous variables such as the patient age will be calculated for counts, means, standard deviations, medians, minimum and maximum values, as well as interquartile ranges. A t-test will be used for data that conform to a normal distribution; if the normal distribution assumption is not met, nonparametric tests will be applied. For categorical data such as sex, the frequency and proportion will be calculated. Groups will be compared using Pearson’s chi-square test and Fisher’s exact probability test. Inferential statistical results (P values) will be listed as descriptive outcomes.

### 5.8 Imputation of missing data

For the primary and secondary endpoints, any missing data will be imputed using the last observed data point and carried forwards to the final analysis, employing the last observation carried forwards (LOCF) approach.

### 5.9 Analysis of the primary outcome

The conclusions of the efficacy analysis are primarily based on the full analysis set (FAS) and the per-protocol set (PPS). The incidence rate of AEs will be calculated as the number of patients who experienced at least one AE divided by the number of evaluated safety cases. The occurrence rates of serious AEs and other AEs will be compared between groups using Pearson’s chi-square test or Fisher’s exact probability test, and the changes in test of routine blood parameters and liver and kidney function before and after treatment will be described. The effectiveness of treatment between the experimental and control groups will be calculated in terms of the number and percentage of effective cases and compared using chi-square tests. A logistic regression model will be established, with the group and sex as independent variables and achievement of the target as the response variable, to analyse the effects of these factors on the ability to reach the target.

### 15.10 Analysis of secondary outcomes

For quantitative data that conform to a normal distribution, a t-test will be employed. In cases where the data do not adhere to a normal distribution, nonparametric tests will be utilized. Statistical analyses will be conducted on both the intention-to-treat (ITT) set and the per protocol set (PPS). If discrepancies arise between the results of these two datasets, the findings from the ITT set will be regarded as the definitive conclusion.

### 15.11 Analysis of safety outcomes

The safety analysis is based on the SS dataset, which uses qualitative assessments to define distributions and cross-tabulation tables to calculate the number of participants and the proportion of abnormal indicators. The changes in routine blood, urine, and stool tests, as well as liver and kidney function, before and after treatment will be compared in both groups, along with the incidence of adverse effects (AEs) (‘the primary focus is AEs associated with SJDHT treatment’). The correlation between adverse events and the study medication will be assessed using the criteria outlined in the ‘Adverse Drug Reaction Reporting and Monitoring Management Methods’ by the Ministry of Health of the People’s Republic of China in 2011.

### 16 Discussion

This study is a stop&go, multicentre, randomized, parallel-controlled, double-blind, superiority clinical trial conducted in China designed to evaluate the efficacy of SJDHT decoction in patients with mild ICIAM. This study will provide clinical evidence for the prevention and treatment of ICIAM with TCM formulations.

TCM has significant advantages in immune-related diseases and anticancer effects. This study aims to validate the clinical efficacy and safety of SJDHT in patients with mild cases, investigate the TCM diagnosis and treatment plan for the population with mild ICIAM n and the evolution of the pathology, and provide an important reference for the integrated treatment of ICIAM using traditional Chinese and Western medicine. This study has many patient resources and a solid research team, with innate advantages that facilitate the vigorous advancement of the research. If the efficacy is superior, SJDHT decoction will be confirmed to be not only effective for symptom management in mild ICIAM but also presumably effective for diseases with the same syndrome and cardiac involvement. This conclusion depends on future comprehensive, multisource, high-quality data, including randomized controlled trials and observational studies.

ICIAM progresses rapidly, and some patients may have a very short mild stage, which can further progress within hours or days. For a large-scale clinical study of patients with a rare disease, accurately targeting this stage and including more potential patients may become significant challenges. This condition requires careful clinical observation and precise syndrome differentiation; after some patients are treated, with increasing experience, the gradual emergence of the pathophysiology and diagnostic markers of this disease will become useful tools to solve this problem.

We must acknowledge that the normalization of biochemical markers does not always equate to clinical recovery, and troponin alone cannot fully reflect the functional status of the myocardium. Consequently, utilizing the time to troponin normalization as the primary endpoint in this study may slightly limit the comprehensiveness of our conclusions. However, compared with other laboratory metrics such as creatine kinase or imaging modalities like echocardiography and magnetic resonance imaging, troponin assessment is more convenient and objective. Furthermore, it demonstrates high representativeness, presenting as abnormal in the vast majority (95%) of the ICIAM patient population (Wang et al., 2023). Additionally, the active components of the SJDHT decoction in this study remain unclear, leading to a certain lack of transparency in the trial. In subsequent research, we aim to elucidate its active constituents through animal experiments and clinical blood sample analyses.

During the research process, we also aim to achieve the following goals: to determine the possibility, success rate, and key points of resuming ICIs during anticancer treatment by improving immune treatment side effects with TCM; to improve tumour immunotherapy with strong side effects and effectively extend the duration of immunotherapy; and to better improve tumour-related symptoms. Moreover, based on real-world data, efforts will be made to fully collect and dynamically observe characteristics of this syndrome in the mild stage and use systems biology and bioinformatics technologies (such as peripheral blood single-cell sequencing) to explore key biomarkers, elucidate the underlying mechanisms of action of SJDHT, combine TCM syndrome elements, construct risk early warning models, and conduct clinical validation to optimize prevention and treatment systems, achieving early intervention and a combination of prevention and treatment.
