# Supplementary material for: Efficacy and safety of Songjiao Dihuang Tang decoction for the dynamic/adaptive treatment of immune checkpoint inhibitor-associated myocarditis: study protocol and statistical analysis plan for a stop&go, multicentre, randomized, parallel-controlled, double-blind, superiority clinical trial
Source: Front Pharmacol. 2026 May 12;17:1797368. doi: 10.3389/fphar.2026.1797368 (PMC13201219; doi:10.3389/fphar.2026.1797368)
Supplement: Supplementary file 1 [file Supplementaryfile5.pdf]

Clinical Research Ethics Committee of China-Japan Friendship Hospital

Ethical Review Approval

**Ethics Review Number**	2024-KY-359
Project full name	Therapeutic Strategies and Evidence-based Research of Traditional Chinese Medicine Regimens in Subclinical and Mild Populations of ICIAM
Sponsor/CRO	China-Japan Friendship Hospital/NA
Research category	Drug clinical trial<br>Clinical trial of medical devices<br>Diagnostic reagent<br>√ Return to clinical research
Project source/Responsible unit	Science and Technology Innovation 2030 — Major Project on "Research on Prevention and Treatment of Cancer, Cardiovascular and Cerebrovascular, Respiratory, and Metabolic Diseases" / China-Japan Friendship Hospital
Principal Investigator /Department	Zheng Jiabin/Oncology Department of Integrative Medicine
Review date	2024/10/17
Review location	Online Meeting
Ethics Committee Review Document	1. Initial Review Application Form<br>2. Curriculum Vitae of the Principal Investigator<br>3. List of Research Members and Table of Division of Duties<br>4. Investigator’s Commitment Letter<br>5. Certificate of Research Project Approval<br>6. Research Protocol (Version No.: 1.0; Version Date: 2024-09-17)<br>7.1-Subclinical Informed Consent Form (Version No.: 1.0; Version Date: 2024-09-17)<br>7.2-Mild Case Informed Consent Form (Version No.: 1.0; Version Date: 2024-09-17)<br>8. Case Report Form (Version No.: 1.0; Version Date: 2024-09-17)<br>9. Current Drug Manufacturing License<br>10. Business License
**Ethics review**	Meeting Review

Review comments

In accordance with the ethical principles of China’s Measures for the Ethical Review of Biomedical Research Involving Humans (2016), Measures for the Administration of Clinical Application of Medical Technologies (2018), Guiding Principles for the Ethical Review of Drug Clinical Trials (2010), Good Clinical Practice for Drug Trials (2020), and Good Clinical Practice for Medical Device Clinical Trials (2022), as well as ICH-GCP, the WMA Declaration of Helsinki, and the CIOMS International Ethical Guidelines for Health-related Research Involving Humans (2016), etc., upon review by this Ethics Committee:

Approval is granted to conduct the clinical study "Therapeutic Strategies and Evidence-based Research of Traditional Chinese Medicine Regimens in Subclinical and Mild Populations of ICIAM" in accordance with the clinical research protocol.

Annual/Regular Follow-up Review Date	Submit annual/regular follow-up review (follow-up frequency: 12 months) 1 month before 2025/10/17		
Approval validity period	2025/10/17	Contact number	010-84206250
Signature of chairperson/vice-chairperson		Approval date	2024/10/18

Clinical Research Ethics Committee of China-Japan Friendship Hospital (Seal)

Statement：

Validity of Approval: This study shall be implemented within 1 year after approval; if it is not implemented within this period, this approval shall automatically become invalid.

The responsibilities, composition, standard operating procedures, and records of the Ethics Committee of China-Japan Friendship Hospital adhere to the ethics review principles of GCP promulgated by China's CFDA and ICH GCP, and comply with the provisions of relevant laws and regulations in China.

"Approved" studies shall be conducted in accordance with the protocol approved by this Ethics Committee and shall comply with the principles of GCP and the Declaration of Helsinki.

For research protocols receiving "Disapproval" or "Suspension or Termination," the sponsor and the investigator may submit a written appeal regarding the issues mentioned in the review opinions and suggestions, stating the reasons. This Ethics Committee may conduct a re-review based on the appeal.

For suspended studies, if there is an intention to resume, an application (application for re-review) must be submitted to the Ethics Committee within 6 months from the date of suspension; the study may only resume after approval by the Ethics Committee. Applications will no longer be accepted after 6 months.

During the research process, any modification made to the research protocol, informed consent form, or recruitment materials, or any change of the Principal Investigator, must be submitted as an "Application for Amendment Review" and may only be implemented after the Ethics Committee reviews and grants approval.

For safety events occurring during the study, the investigator shall report them promptly to this Ethics Committee for review in accordance with GCP requirements.

Serious protocol violations or persistent protocol violations occurring at this center shall be reported promptly to this Ethics Committee for review.

Annual/Periodic Research Progress Report: The investigator/sponsor shall submit a follow-up review report 1 month prior to the specified date, based on the frequency and date of follow-up reviews.

Upon completion of the study, please submit a Study Completion Report and a Center Summary Table to the Ethics Committee.
