# Supplementary material for: Efficacy and safety of Songjiao Dihuang Tang decoction for the dynamic/adaptive treatment of immune checkpoint inhibitor-associated myocarditis: study protocol and statistical analysis plan for a stop&go, multicentre, randomized, parallel-controlled, double-blind, superiority clinical trial
Source: Front Pharmacol. 2026 May 12;17:1797368. doi: 10.3389/fphar.2026.1797368 (PMC13201219; doi:10.3389/fphar.2026.1797368)
Supplement: Supplementary file 2 [file Supplementaryfile1.pdf]

China Isotope & Radiation Corporation Beijing Huamiao Pharmaceutical Co., Ltd.

Testing Report of botanical and animal-derived drugs

Report number: C2025052916

Sample name	Shuiniujiao (slices)	Product ID	C2025052202
Lot number	750516101	Place of production	Baise, Guangxi
Quantity	193.00kg	Specifications	Slice
Packing specifications	--	Sampling period	22 May 2025
Inspecting item	Complete inspection	Report date	29 May 2025
Report basis	Pharmacopoeia of the People's Republic of China (2020 Edition, Volume I)		

Inspecting Item	Standard Code	Result
[shape and properties]	This product should be in the form of irregular shreds or slices. The surface is brownish-black or greyish-black, with fine, straight textures, and sometimes exhibits streaks of varying shades. The texture is tough and elastic. The odor is slightly fishy, and the taste is mild.	This product should be in the form of irregular shreds or slices. The surface is brownish-black or greyish-black, with fine, straight textures, and sometimes exhibits streaks of varying shades. The texture is tough and elastic. The odor is slightly fishy, and the taste is mild.
[Identification] MiCroscopic	Should exhibit the microscopic characteristics as specified in the standard.	Exhibits the microscopic characteristics as specified in the standard.
[Examination] Sulfur dioxide residues	Should not exceed 150 mg/kg	76.4mg/kg
Foreign matter	Should not exceed 3%	0%
Water	Should not exceed 13%.	11%

Test Conclusion: This product was tested in accordance with the Pharmacopoeia of the People's Republic of China (2020 Edition, Volume I); the results comply with the requirements.

Authorized signatory: 王艳燕

Date of issue: 2025-5-29

China Isotope & Radiation Corporation Beijing Huamiao Pharmaceutical Co., Ltd.

Testing Report of botanical and animal-derived drugs

Report number: C2025071409

Sample name	Yousongjie	Product ID	C2025070901
Lot number	750706406	Place of production	Baoding, Hebei
Quantity	141.00kg	Specifications	Block
Packing specifications	--	Sampling period	09 July 2025
Inspecting item	Complete inspection	Report date	14 July 2025
Report basis	Pharmacopoeia of the People's Republic of China (2020 Edition, Supplement I)		

Inspecting Item	Standard Code	Result
[shape and properties]	This product should be in the form of irregular thin slices or blocks of varying sizes. The outer surface is yellow-brown, grey-brown, or reddish-brown. The body is relatively heavy and the texture is hard. It has a turpentine-like aroma, with a slightly bitter and pungent taste.	This product should be in the form of irregular thin slices or blocks of varying sizes. The outer surface is yellow-brown, grey-brown, or reddish-brown. The body is relatively heavy and the texture is hard. It has a turpentine-like aroma, with a slightly bitter and pungent taste.
[Examination]		
Sulfur dioxide residues	Shall not exceed 150 mg/kg	0mg/kg
Foreign matter	Shall not exceed 3%	0%
Water	Shall not exceed 9.0%	7.4%
[Extractives]	Shall not be less than 22.0%	22.9%

Test Conclusion: This product was tested in accordance with the Pharmacopoeia of the People's Republic of China (2020 Edition, Supplement I); the results comply with the requirements.

Remarks: The data for sulfur dioxide residues under the [Examination] item are cited from the data of Yousongjie (Batch No. Y75052641).

Authorized signatory: [illegible]   Date of issue: 2025-7-14

China Isotope & Radiation Corporation Beijing Huamiao Pharmaceutical Co., Ltd.

Testing Report of botanical and animal-derived drugs

Report number: C2025071405

Sample name	Dihuang	Product ID	C2025070123
Lot number	750616106	Place of production	Jiaozuo, Henan
Quantity	980.00kg	Specifications	Sheet
Packing specifications	--	Sampling period	01 July 2025
Inspecting item	Complete inspection	Report date	14 July 2025
Report basis	Pharmacopoeia of the People's Republic of China (2020 Edition, Volume I)		

**Inspecting Item**	**Standard Code**	**Result**
[shape and properties]	This product should be in the form of circular or curled thin slices. For Moutan Cortex with cork (Liandanpi), the outer surface is greyish-brown or yellowish-brown, and pink where the cork has exfoliated; for Moutan Cortex without cork (Guadanpi), the outer surface is reddish-brown or pale greyish-yellow. The inner surface sometimes exhibits shiny crystals. The cut surface is pale pink and starchy. Odor, aromatic; taste, slightly bitter and astringent.	This product is in the form of circular or curled thin slices. For Guadanpi, the outer surface is reddish-brown or pale greyish-yellow. The inner surface sometimes exhibits shiny crystals. The cut surface is pale pink and starchy. Odor, aromatic; taste, slightly bitter and astringent.
[Identification] MiCroscopic	Should exhibit the microscopic characteristics as specified in the standard.	Exhibits the microscopic characteristics as specified in the standard.
TLC Identification 1	In the chromatogram of the test solution, spots of the same color should be observed at positions corresponding to the chromatogram of the catalpol reference substance solution.	In the chromatogram of the test solution, spots of the same color are observed at positions corresponding to the chromatogram of the catalpol reference substance solution.
TLC Identification 2	In the chromatogram of the test solution, spots of the same color should be observed at positions corresponding to the chromatogram of the verbascoside reference substance solution.	In the chromatogram of the test solution, spots of the same color are observed at positions corresponding to the chromatogram of the verbascoside reference substance solution.
[Examination]		
Sulfur dioxide residues	Shall not exceed 150 mg/kg	0mg/kg
Foreign matter	Shall not exceed 3%	0%
Water	Shall not exceed 15%	11%
Total ash	Shall not exceed 8%	3.9%
Acid-insoluble ash	Shall not exceed 3%	0.74%
[Extractives]	Shall not be less than 65%	88.7%
[Assay]		
Assay 1	Rehmanniae Radix contains catalpol($C_{15}H_{22}O_{10}$) shall not be less than 0.20%	0.41%
Assay 2	Rehmanniae Radix contains rehmannioside D($C_{27}H_{42}O_{20}$) shall not be less than 0.10%	0.16%

China Isotope & Radiation Corporation Beijing Huamiao Pharmaceutical Co., Ltd.

Testing Report of botanical and animal-derived drugs

Report number: C2025071405

Sample name	Dihuang	Product ID	C2025070123
Lot number	750616106	Place of production	Jiaozuo, Henan
Quantity	980.00kg	Specifications	Sheet
Packing specifications	--	Sampling period	01 July 2025
Inspecting item	Complete inspection	Report date	14 July 2025
Report basis	Pharmacopoeia of the People's Republic of China (2020 Edition, Volume I)		

Test Conclusion: This product was tested in accordance with the Pharmacopoeia of the People's Republic of China (2020 Edition, Volume I); the results comply with the requirements.

Remarks: The data for sulfur dioxide residues under the [Examination] item are cited from the data of Dihuang (Batch No. Y75021811).

Authorized signatory: 王艳 [illegible]

Date of issue: 2025-7-14

China Isotope & Radiation Corporation Beijing Huamiao Pharmaceutical Co., Ltd.
Testing Report of botanical and animal-derived drugs

Report number: C2025070901

Sample name	Baishao	Product ID	C2025070119
Lot number	750619504	Place of production	Bozhou, Anhui
Quantity	5950.00kg	Specifications	Slice
Packing specifications	--	Sampling period	01 July 2025
Inspecting item	Complete inspection	Report date	09 July 2025
Report basis	Pharmacopoeia of the People's Republic of China (2020 Edition, Volume I),National Compilation of Processing Norms for Chinese Materia Medica Decoction Pieces		

**Inspecting Item**	**Standard Code**	**Result**
[shape and properties]	The specimen shall present as sub-circular thin slices. The surface is light brownish-red or sub-white. The cut surface is slightly brownish-red or sub-white, featuring a distinct cambium ring and slightly elevated vascular bundles arranged in a radial pattern. It possesses a slight odor and a slightly bitter, sour taste.	The specimen shall present as sub-circular thin slices. The surface is light brownish-red or sub-white. The cut surface is slightly brownish-red or sub-white, featuring a distinct cambium ring and slightly elevated vascular bundles arranged in a radial pattern. It possesses a slight odor and a slightly bitter, sour taste.
[Identification] MiCroscopic	Should exhibit the microscopic characteristics as specified in the standard.	Exhibits the microscopic characteristics as specified in the standard.
TLC Identification	In the chromatogram of the test preparation, blue -purple spots shall be exhibited at locations corresponding to those in the chromatogram of the paeoniflorin reference standard.	In the chromatogram of the test preparation, blue-purple spots are exhibited at locations corresponding to those in the chromatogram of the paeoniflorin reference standard.
[Examination]		
Sulfur dioxide residues	Shall not exceed 400 mg/kg	291mg/kg
Foreign matter	Shall not exceed 3%	0%
Water	Shall not exceed 14%	10.8%
Total ash	Shall not exceed 4%	2.8%
[Extractives]	Shall not be less than 22%	27.9%
[Assay]		
Assay	Calculated on the dried basis, this product contains not less than 1.2% of paeoniflorin ($C_{23}H_{28}O_{11}$).	2.3%

Test Conclusion: This product was tested in accordance with the Pharmacopoeia of the People's Republic of China (2020 Edition, Volume I) and the National Processing Standards for Chinese Materia Medica Decoction Pieces; the results comply with the specifications.

Authorized signatory: [illegible]

Date of issue: 2025-7-9

China Isotope & Radiation Corporation Beijing Huamiao Pharmaceutical Co., Ltd.

Testing Report of botanical and animal-derived drugs

Report number: C2025082904

Sample name	Mudanpi	Product ID	C2025081910
Lot number	750731504	Place of production	Bozhou, Anhui
Quantity	5915.50kg	Specifications	Slice
Packing specifications	--	Sampling period	19 August 2025
Inspecting item	Complete inspection	Report date	29 August 2025
Report basis	Pharmacopoeia of the People's Republic of China (2020 Edition, Volume I)		

Inspecting Item	Standard Code	Result
[shape and properties]	This product occurs as circular or curled thin slices. For the unscraped pieces, the outer surface is greyish-brown or yellowish-brown, and pink where the cork has exfoliated; for the scraped pieces, the outer surface is reddish-brown or pale greyish-yellow. The inner surface occasionally shows shiny crystals. The cut surface is pale pink and starchy. Odour, aromatic; taste, slightly bitter and astringent.	This product occurs as circular or curled thin slices. The outer surface of the scraped pieces is reddish-brown or pale greyish-yellow. The inner surface occasionally shows shiny crystals. The cut surface is pale pink and starchy. Odour, aromatic; taste, slightly bitter and astringent.
[Identification] MiCroscopic	Should exhibit the microscopic characteristics as specified in the standard.	Exhibits the microscopic characteristics as specified in the standard.
TLC Identification	In the chromatogram of the test solution, spots of the same color shall be exhibited at the positions corresponding to the chromatogram of the paeonol reference standard.	In the chromatogram of the test solution, spots of the same color are exhibited at the positions corresponding to the chromatogram of the paeonol reference standard.
[Examination]		
Sulfur dioxide residues	Shall not exceed 150 mg/kg	89.5mg/kg
Foreign matter	Shall not exceed 3%	0%
Water	Shall not exceed 13%	9.25%
Total ash	Shall not exceed 5%	4.7%
[Extractives]	Shall not be less than 15%	25.7%
[Assay]		
Assay	Calculated on the dried basis, this product contains not less than 1.2% of paeonol($C_9H_{10}O_3$).	2.8%

Test Conclusion: This product was tested in accordance with the Pharmacopoeia of the People's Republic of China (2020 Edition, Volume I); the results comply with the specifications.

Authorized signatory:

Date of issue:2025-8-29
