# Supplementary material for: Efficacy and safety of Songjiao Dihuang Tang decoction for the dynamic/adaptive treatment of immune checkpoint inhibitor-associated myocarditis: study protocol and statistical analysis plan for a stop&go, multicentre, randomized, parallel-controlled, double-blind, superiority clinical trial
Source: Front Pharmacol. 2026 May 12;17:1797368. doi: 10.3389/fphar.2026.1797368 (PMC13201219; doi:10.3389/fphar.2026.1797368)
Supplement: Supplementary file 5 [file Supplementaryfile3.pdf]

清華大學

药学技术中心

Center of Pharmaceutical Technology

测试报告

**研究名称：**	中药复方化学成分及入血成分检测
**送样人信息：**	中日友好医院郑佳彬
**检测负责单位信息：**	清华大学药学院药学技术中心（盖章）
	中国 北京 100084
	北京市海淀区清华大学郑裕彤医学楼 E206
	电话：(010)62788677
**检测人：**	田国芳
**检测单位联系人：**	唐煜 邮箱：tangyu@mail.tsinghua.edu.cn
	电话：(010)62795450
**报告日期：**	2025 年 11 月 25 日

目录

1. 实验目的 3
2. 样品信息 3
3. 实验仪器和试剂 3
4. 实验条件 3
5. 实验结果 4

1.实验目的

采用Waters Synapt G2-Si Qtof高分辨质谱及Unifi软件进行中药化学成分及入血成分检测。

2.样品信息

对于中药样品，取样品200mg，置于15mL 离心管内，加入10ml50%甲醇水溶液（v：v，甲醇：水=50：50），混匀，取上清 1mL 置于离心管中，14000rpm 离心 5min，取上清过 0.22um 微孔滤膜后，置入进样瓶中，待 UHPLC-MS/MS 分析。空白样品采用相同条件处理。

对于血浆样品，取血浆样品 100μL，置于 1.5mL EP 管内，加入 500μL 乙腈沉淀蛋白，涡旋 2min，14000rpm 离心 5min，取上清液 500μL，氮气吹干，用 100μL 50%乙腈水溶液复溶，置入进样瓶中，待 UHPLC-MS/MS 分析。空白血浆样品采用相同条件处理。

3.实验仪器和试剂

3.1 实验仪器和设备

名称	型号	供应商
Waters Synapt G2-Si Qtof 质谱仪	Synapt G2-Si	Waters
ACQUITY UPLC I Class system 液相色谱	I Class	Waters
涡旋混合器	Vortex-2 Genie	Scientific Industries
低温离心机	5810R	Eppendorf
超声波清洗器	WD-9415C	北京市六一仪器厂
色谱柱	ACQUITY UPLC HSS T3，2.1×100mm，1.8μm	Waters

3.2 试剂

甲醇，甲酸购自 Thermo Fisher 公司；去离子水，制备自 MilliQ Advantage A10 超纯水机；1.5mL 离心管、15mL 离心管及进样小瓶均购自 Axygen 公司。

4.实验条件

4.1 液相条件

色谱柱：ACQUITY UPLC HSS T3 column（2.1×100mm，1.8μm）

柱温：35°C

进样体积：10μL

流速：0.25mL/min

流动相：A（去离子水，含 0.1%甲酸）；B（乙腈，含 0.1%甲酸），梯度洗脱。具体梯度洗脱条件如下：

Time	FlowRate（mL/min）	%A	%B
0	0.25	100	0
15	0.25	80	20
50	0.25	0	100
60	0.25	0	100
70	0.25	100	0

4.2 质谱条件

Project	Condition
质谱系统	Synapt G2-Si
质量范围	50-1500Da
扫描时间	0.1s
扫描模式	MSe, ESI- and ESI+ in resolution mode
参比化合物	Leucine Enkephalin(LE) 1ppm (scanfor0.3s,interval15s)
毛细管电压	3kV(ESI+)/2.5kV(ESI-)
锥孔电压	40V
碰撞能(eV)	10-50eV
离子源温度	125℃
脱溶剂温度	500℃
锥孔气流速	50L/H
脱溶剂气流速	800L/H
分析时间	70min

5.实验结果

5.1 总离子流图(TIC)

中药复方样品正离子模式和负离子模式下的 TIC 扫描图分别如**图 1** 和**图 2** 所示。

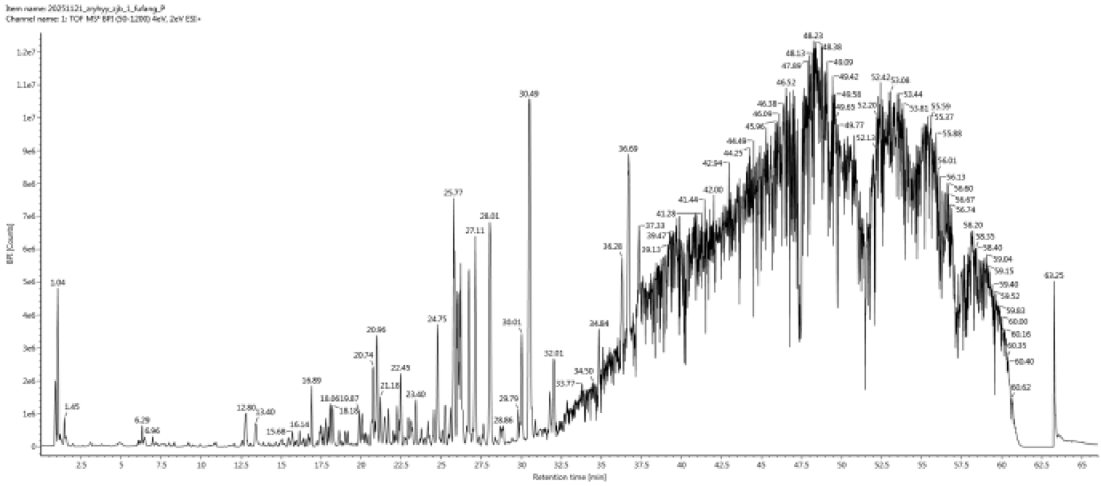

图 1 中药复方的正离子模式 TIC 图

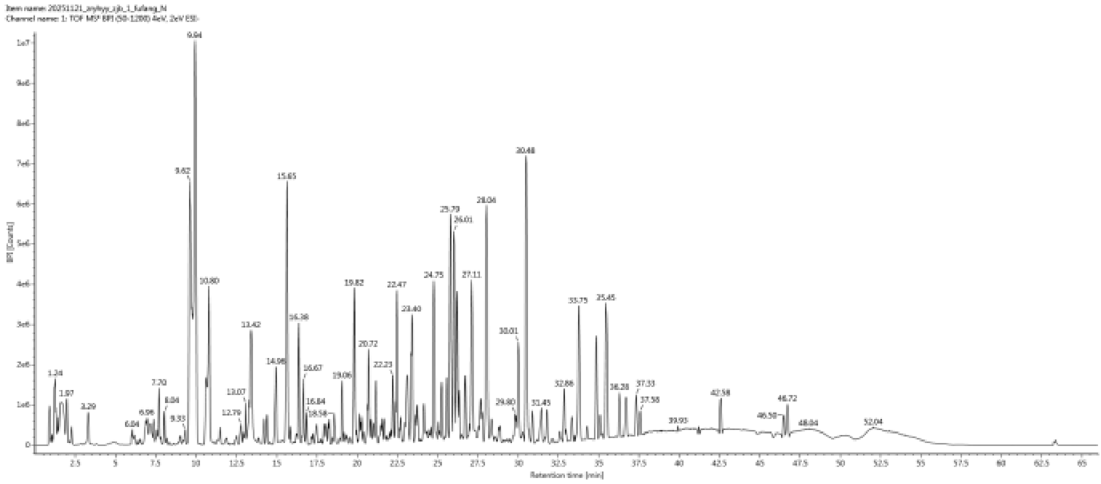

图 2 中药复方的负离子模式 TIC 图

给药后血浆样品正离子模式和负离子模式下的 TIC 扫描图分别如**图 3** 和**图 4** 所示。

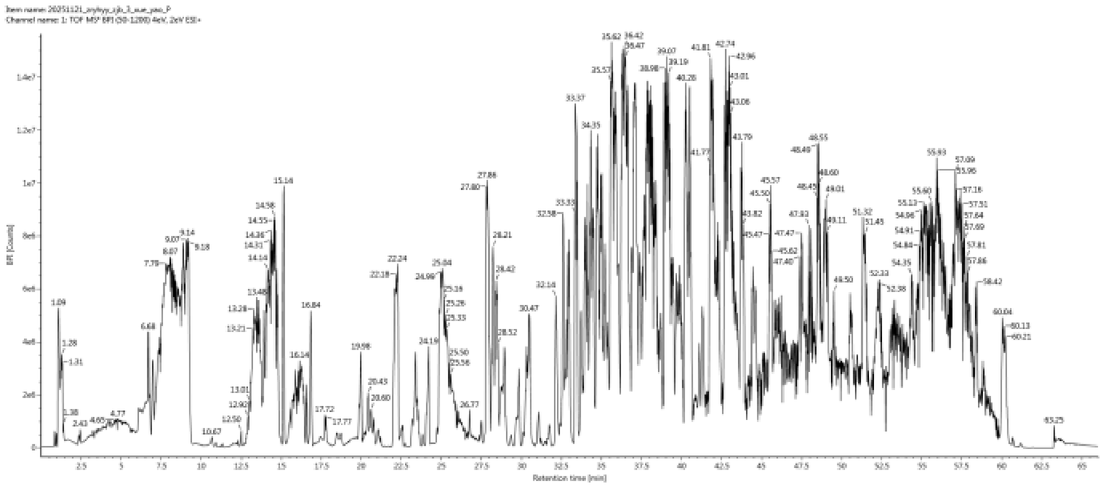

图 3 给药血浆样品的正离子模式 TIC 图

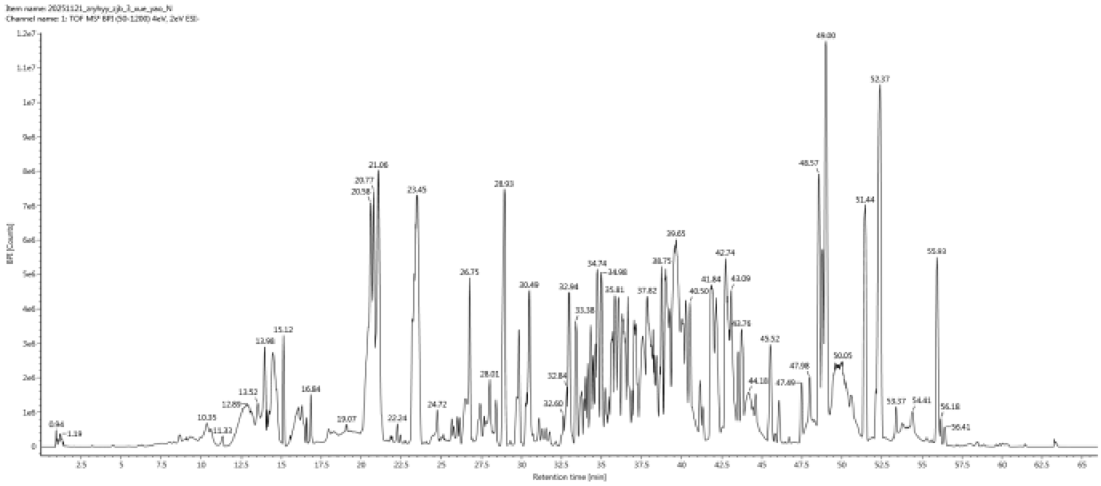

图 4 给药血浆样品的负离子模式 TIC 图

空白血浆样品正离子模式和负离子模式下的 TIC 扫描图分别如**图 5** 和**图 6** 所示。

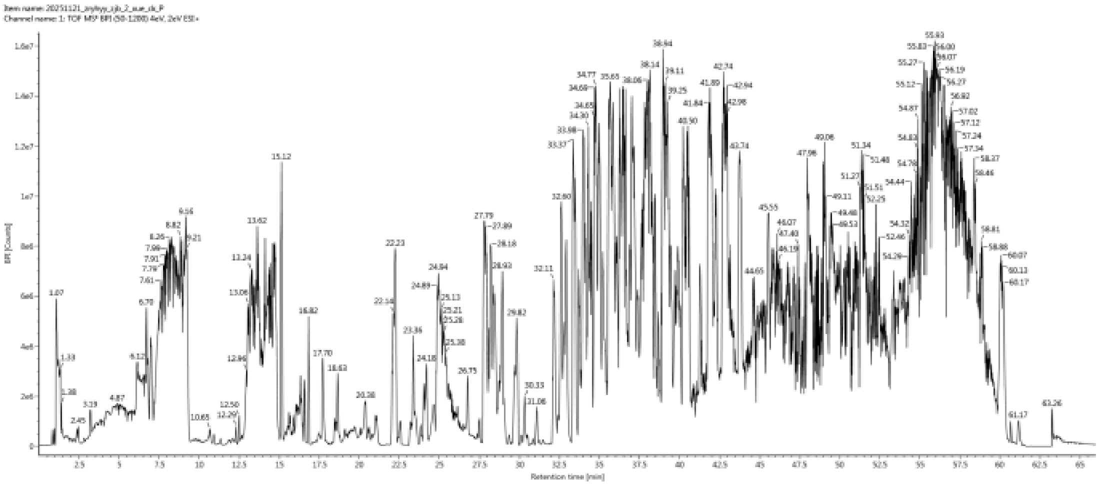

图 5 空白血浆样品的正离子模式 TIC 图

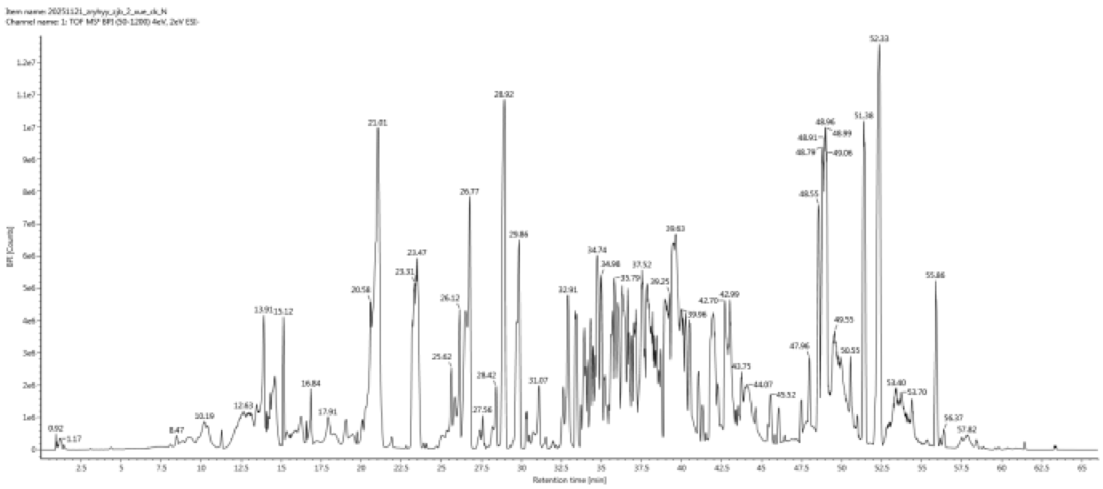

图 6 空白血浆样品的负离子模式 TIC 图

5.2 中药化学成分鉴定结果

采用 UPLCI-Class 和 SynaptG2-SiQtof 用于中药化学成分的色谱分离和质谱数据采集，结合 Unifi 软件中天然产物的整体工作流程，基于 6400 天然产物的理论质谱数据库对产品成分进行数据处理，结果如表 1 所示。

表 1 中药复方化合物鉴定信息

见数据表“TCM 结果”

5.3 中药入血成分分析

采用 UPLCI-Class 和 SynaptG2-SiQtof 用于中药化学成分的色谱分离和质谱数据采集，结合 Unifi 软件中天然产物的整体工作流程，基于 6400 天然产物的理论质谱数据库对产品成分进行数据处理。

将空白血浆作为 Reference 参比样品，将给药后血浆作为 Unknown 实验样品，同时采用 Unifi 软件中天然产物的整体工作流程进行化学物质基础分析，将在给药后血浆中存在，并且在空白血浆中不存在或丰度接近基线噪音的化合物认为是潜在的入血成分，具体入血成分分析结果如表 3 所示大部分化合物在空白血浆中未见信号响应，且在中药复方的检测中存在。

化合物的 MS^1 谱图、MS^2 谱图、提取离子流图、结构式及在空白样品和给

药样品中的峰面积比较详见附表。

表 3 给药后血浆样品的入血成分信

见数据表"入血结果"
