# Supplementary material for: Efficacy and safety of Songjiao Dihuang Tang decoction for the dynamic/adaptive treatment of immune checkpoint inhibitor-associated myocarditis: study protocol and statistical analysis plan for a stop&go, multicentre, randomized, parallel-controlled, double-blind, superiority clinical trial
Source: Front Pharmacol. 2026 May 12;17:1797368. doi: 10.3389/fphar.2026.1797368 (PMC13201219; doi:10.3389/fphar.2026.1797368)
Supplement: Supplementary file 6 [file Supplementaryfile6.pdf]

Informed consent form (Mild Case Group)

Subject Information Page

Protocol Title: Treatment strategies and evidence-based research of Traditional Chinese Medicine (TCM) solutions in ICIAM subclinical and mild populations

Principal Investigator: Zheng Jiabin

Sponsor: China-Japan Friendship Hospital

Project Source: Prevention and treatment research of cancer, cardiovascular, cerebrovascular, respiratory, and metabolic diseases

Dear Participant,

You are invited to participate in the research study titled **"Treatment strategies and evidence-based research of TCM solutions in ICIAM subclinical and mild populations,"** supported by **the China-Japan Friendship Hospital**. Please read this informed consent form carefully and make a prudent decision regarding your participation. Participation is entirely voluntary. You must provide written consent before joining. You may ask the study doctor or staff to explain any parts you do not understand, and we encourage you to discuss this with family and friends. You have the right to refuse participation or withdraw at any time without penalty or loss of benefits. If you are participating in other research, please inform the staff:

Research Background

Immune Checkpoint Inhibitor-Associated Cardiac Injury (ICIACI) has a low incidence (0.06% – 3.8%) but a very high mortality rate (39.7% – 66%). Despite existing guidelines, clinical outcomes remain poor due to a lack of effective treatments. TCM management for immune-related adverse events (irAEs) is currently being explored. Preliminary research has shown the unique advantages of TCM in this area and established a multi-center clinical database. This study aims to determine if TCM can intercept disease progression and reduce mortality, providing high-quality, internationally recognized evidence.

Research Objectives

To establish a management platform for mild ICIAM cases using integrated Chinese and Western medicine and verify the TCM pathogenesis evolution from "toxin damaging the heart body" to "heat scorching yin injury".

To conduct a multi-center randomized controlled trial (RCT) to verify the clinical efficacy and safety of the "Songjiao Dihuang Decoction" in mild cases.

To explore the biological nature of ICIAM progression and discover TCM diagnostic and therapeutic biomarkers.

Research Process

I. Participants

Approximately 200 people will participate, with a 1:1 ratio between the experimental and control groups. China-Japan Friendship Hospital will enroll 100 cases.

II. Methodology

If you agree to participate in this study, please sign this Informed Consent Form. This study is a randomized, double-blind, controlled trial that utilizes an Interactive Web Response System (IWRS) and SAS-based stratified block randomization. Participants will be assigned to either the experimental group or the control group in a 1:1 ratio through competitive enrollment. Unblinding will take place after the database

is locked; however, emergency unblinding will be performed should a medical emergency occur. The specific study procedures are as follows:

1. Screening Period

Before you are enrolled in the study, the doctor will inquire about and record your medical history. A comprehensive physical examination will be completed within 7 days prior to starting the study medication Laboratory tests will be conducted, including a complete blood count (CBC), blood biochemistry, the four cardiac biomarkers, cytokines, and lymphocyte subsets. Additionally, examinations such as an echocardiogram (Echo) and a 2-lead electrocardiogram (ECG) will be performed. You will also be required to complete medical history collection, quality-of-life questionnaires, and Traditional Chinese Medicine (TCM) syndrome scoring scales.

2.Treatment Intervention

During the mild stage of the disease, you will receive either Songjiao Dihuang Formula granules (consisting of Buffalo Horn, Rehmannia root, Tree Peony bark, Pine node, Agrimony, etc.) provided by Guangdong Yifang (investigational drug) or a placebo (10% Songjiao Dihuang Decoction) (investigational drug). The placebo is designed to be similar to the experimental Songjiao Dihuang granules in characteristics such as color, odor, taste, shape, and texture, making it difficult for both researchers and participants to distinguish between the two. The dosage is 10 mg per administration, taken twice daily (Bid). Medication will begin on Day 1 of enrollment and, following the initial assessment, will continue for an additional 4 weeks. Methylprednisolone will be administered according to the treatment pathway, and you will be observed and followed up until your cardiac troponin (cTn) levels return to baseline. If cTn levels increase by >50% at the first follow-up, the corticosteroid dosage will be increased and other intensified medical treatments (such as JAK inhibitors) will be provided. Following 48 – 72 hours of treatment, a re-evaluation will be conducted. If symptoms worsen or cTn levels increase by >50% again, you may enter the severe case study or withdraw from the study based on the researcher's medical opinion and your own preference.

During the period of medication, a comprehensive physical examination and routine laboratory tests—including complete blood count (CBC), blood biochemistry, the four cardiac biomarkers, echocardiogram, and 12-lead electrocardiogram (ECG)—will be completed every 2 weeks. Additionally, tests for cytokines and lymphocyte subsets will be conducted every 4 weeks.

During the EOT visits at the 4th and 6th months, a comprehensive physical examination and routine laboratory tests (including complete blood count, blood biochemistry, the four cardiac biomarkers, echocardiogram, 12-lead ECG, cytokines, and lymphocyte subsets) will be completed every 2 months.

A safety follow-up will be conducted 30 days (±7 days) after the EOT visit. Safety reporting will continue for at least 30 days following the last dose, or until toxicity resolves, returns to baseline, or is deemed irreversible. The survival follow-up period will begin 60 days (±7 days) after the safety follow-up ends. Subsequent survival follow-ups will occur at a frequency of once every 60 days (±7 days) until the subject's death, loss to follow-up, study termination, or other study-end criteria are met.

The laboratory tests mentioned above (complete blood count, blood biochemistry, four cardiac biomarkers, cytokines, and lymphocyte subsets) and examinations (echocardiogram and 12-lead ECG) are all part of the routine diagnosis and treatment process. Standard glucocorticoid therapy is also considered routine care. The Songjiao Dihuang Formula granules (or placebo) are the specific interventions for this study and will be provided to you free of charge.

3. How long will this study last?

For the mild stage, the medication starts on Day 1 of enrollment. Following the initial assessment, the treatment will continue for an additional 4 weeks. A re-evaluation will be conducted after 48–72 hours of treatment, followed by a 6-week follow-up period, totaling approximately 10 weeks.

You may choose to withdraw from the study at any time without losing any benefits to which you are otherwise entitled. However, if you decide to withdraw during the study, we encourage you to consult with your doctor first. If you experience a serious adverse event, or if your study doctor determines that continuing in the study is not in your best interest, he/she will decide to withdraw you from the study. The sponsor or regulatory authorities may also terminate the study during the research period. Your withdrawal will not affect your normal medical treatment, and your rights and interests will remain unaffected.

If you withdraw from the study for any reason, you may be asked about your participation. If the doctor deems it necessary, you may also be requested to undergo laboratory tests and physical examinations.

4. Information and Biological Samples Collected in the Study

Biological samples collected during the study, such as blood, urine, and stool, will be used for routine diagnosis and treatment. These samples will be managed through anonymization, combined with strict management and supervision of staff and visitors to prevent data leakage and infringement.

Your personal and medical information will be used for scientific research purposes only and will not be used for any other purposes. We will establish dedicated Case Report Forms (CRFs), which will only display your identification number and will not include your real name, phone number, home address, or other personal identifiers. At the same time, we will properly record, process, store, and maintain the confidentiality of your clinical information. In the future, the preserved information and/or samples may be utilized for secondary research, such as retrospective analysis of the relationship between the disease and treatment, or analysis of liver and kidney function through blood samples.

Risks and Benefits

1.What are the risks of participating in this study?

The risks of participating in this study are listed below. You should discuss these risks with your study doctor or, if you prefer, with your regular primary care physician.

Participants in both groups may experience side effects associated with immunosuppressants such as corticosteroids, including but not limited to: nausea, vomiting, bloody stools, secondary infections, impaired liver function, hyperglycemia (high blood sugar), and avascular necrosis of the femoral head. If any of these adverse reactions are severe, treatment should be discontinued, and appropriate symptomatic treatment will be provided. Potential adverse reactions from taking Songjiao Dihuang Formula granules (or the placebo) may include allergies, nausea, vomiting, and diarrhea. If such adverse reactions occur, the medication will be stopped, and symptomatic treatment will be administered.

Furthermore, we will make every effort to protect the information you provide from disclosure; however, we cannot guarantee the absolute security of your information. Some questions we ask during the study may make you feel uncomfortable; you may refuse to answer such questions, and you are welcome to take a break at any time during the process. You may withdraw from this study at any moment.

If you experience any discomfort, new changes in your condition, or any unexpected circumstances during the study—regardless of whether they are related to the research—you must notify your doctor immediately. He or she will make a clinical judgment and provide appropriate medical treatment.

During the study, you will need to visit the hospital for scheduled follow-ups and examinations. This will require some of your time and may cause trouble or inconvenience.

2.What are the benefits of participating in this study?

Direct Benefits: If you agree to participate in this study, you may receive direct medical benefits, and your condition may improve; however, we cannot guarantee that you will receive such benefits.

Potential Benefits: We hope that the information obtained from your participation in this study will benefit you or future patients with the same medical condition.

Alternative Treatment Options

Apart from participating in this study, you may receive the routine treatment provided by your physician, which includes immunosuppressants such as corticosteroids.

Use of Research Results and Confidentiality of Personal Information

We are fully aware of the importance of your personal information, especially within the context of clinical research. We respect and protect the privacy of research participants and commit to strictly complying with legal and regulatory requirements, while referring to established industry security standards, to safeguard the security of your personal information.

With the understanding and cooperation of you and other participants, the results obtained from this research project may be published in medical journals; however, we will maintain the confidentiality of your research records as required by law. The personal information of research participants will be kept strictly confidential, and your personal information will not be disclosed unless required by relevant laws. When necessary, government regulatory departments, the Hospital Ethics Committee, and other relevant research personnel may access your data in accordance with regulations.

Research Expenses and Compensation

I. Costs of Study Medications/Devices and Related Examinations

This study will provide the Songjiao Dihuang Decoction/placebo granules required for treatment free of charge. Immunosuppressants, such as corticosteroids, are required for your routine treatment and will be the responsibility of the patient. Routine laboratory tests and examinations required for your treatment plan (such as blood collection and imaging examinations), as well as routine treatments and examinations required for any other concurrent conditions you may have, are not covered under the free-of-charge provisions of this study.

II. Compensation for Participation

There is no other compensation for participating in this study.

III. Compensation/Indemnity for Study-Related Injury

If a study-related injury occurs, you will receive free medical treatment provided by the China-Japan Friendship Hospital and will be entitled to compensation/indemnity in accordance with relevant Chinese laws.

Participant Rights and Important Considerations

I. Your Rights

Your participation in this study is entirely voluntary throughout the entire process. If you decide not to participate, it will not affect any other treatments you are entitled to receive. If you decide to participate, you will be required to sign this written Informed Consent Form. You have the right to withdraw from the trial at any stage at any time without facing discrimination or unfair treatment; your corresponding medical treatment and rights will remain unaffected.

If you experience a serious adverse reaction during the study, your doctor will examine you and provide symptomatic treatment. If you are unable to tolerate the adverse reactions of the medication or fail to follow the doctor's instructions, the doctor may advise you to withdraw from the study.

If you decide to no longer participate in this study, we hope you will inform your study doctor in a timely manner, so that the study doctor can provide advice and guidance regarding your health status.

Your participation in this study will be discontinued or terminated if any of the following conditions occur: certain complications or comorbidities arise during the clinical trial making it unsuitable to continue; a serious adverse event occurs making it unsuitable to continue; poor compliance that affects the assessment of efficacy and safety; the investigator believes there is a safety reason to prematurely terminate or suspend the clinical research; or you choose to withdraw from the trial for any reason.

For participants who withdraw midway, we have a final follow-up plan for safety considerations; however, you have the right to refuse this. Additionally, we hope you will return all unused study medications to your study doctor. If new information relevant to your health and rights is discovered after your withdrawal, we may contact you again.

II. Important Considerations

As a study participant, you are required to fulfill the following responsibilities:

- Provide truthful information regarding your personal medical history and current physical condition.
- Inform the study doctor of any discomfort or symptoms you discover during the course of this study.
- Do not consume any restricted medications, foods, or other substances as instructed by your doctor.
- At each required visit, please return all unused study medications and all empty packaging to the study doctor.
- Store the study medication in a refrigerator (cold storage); keep the study medication out of the reach of children; and do not give the study medication to anyone else.
- Inform the study doctor whether you have recently participated in other research or are currently participating in any other research.

Contact Information for Obtaining Information

If any important new information arises during the research process that may affect your willingness to continue participating in the study, your doctor will notify you in a timely manner. If you have questions about your own research data or wish to know the findings of this study after its conclusion, you may ask any questions related to this research at any time and receive corresponding answers. Please contact Dr. Zheng Jiabin via telephone at 18618350234.

The Ethics Committee has reviewed and approved this study. If you have any questions related to your rights or interests, or if you wish to report any difficulties, dissatisfaction, or concerns encountered during your participation in this study, or if you wish to provide comments and suggestions related to this research, please contact the Clinical Research Ethics Committee of China-Japan Friendship Hospital.

- Telephone: 010-84206250
- Email: zryyec@126.com

Informed consent form (consent signature page)

Informed Consent Statement

I have been informed of the purpose, background, procedures, risks, and benefits of this study. I have had sufficient time and opportunity to ask questions, and I am satisfied with the answers provided.

I have also been informed of whom to contact if I have questions, wish to report difficulties, concerns, or suggestions regarding the study, seek further information, or provide assistance to the research.

I have read this Informed Consent Form and agree to participate in this study.

I understand that I have the choice not to participate in this study, or to withdraw from this study at any time during the research process without providing any reason.

I am aware that if my condition worsens, if I experience a serious adverse event, or if my study doctor determines that continuing in the study is not in my best interest, he/she will decide to withdraw me from the research. The sponsor or regulatory authorities may also terminate the study during the research period without my consent. Should this occur, the doctor will notify me in a timely manner, and the study doctor will discuss other available options with me.

I will receive a copy of this Informed Consent Form, which includes both my signature and the researcher's signature.

Patient signature: _ _ _ _ _ _ _ _ _ _ _ _ _ _ _ _Date: _ _ _ _ _ _ _ _ _ _ _ _ _ _ _ _

(Note: If the subject is incapacitated or has limited capacity, the signature and signature date of a legal representative are required.)

Patient Contact Information:_ _ _ _ _ _ _ _ _ _ _ _ _ _

Legal Representative Signature: _ _ _ _ _ _ _ _ _ _ _ _ _ _ Date: _ _ _ _ _ _ _ _ _ _ _ _ _ _ _ _

(Note: If the subject is unable to read this Informed Consent Form, an independent witness is required to certify that the investigator has informed the subject of all the contents of the Informed Consent Form. The independent witness must provide their signature and the date of signing.)

Legal Representative Contact Information:_ _ _ _ _ _ _ _ _ _ _ _ _ _

Independent Witness Signature:_ _ _ _ _ _ _ _ _ _ _ _ _Date:_ _ _ _ _ _ _ _ _ _ _ _ _

Independent Witness Contact Information:_ _ _ _ _ _ _ _ _ _ _ _

Investigator Signature: _ _ _ _ _ _ _ _ _ _ _ _Date: _ _ _ _ _ _ _ _ _ _ _ _

Investigator Contact Information:_ _ _ _ _ _ _ _ _ _ _ _
